# Engineering synthetic suppressor T cells that execute locally targeted immunoprotective programs

**DOI:** 10.1126/science.adl4793

**Published:** 2024-12-06

**Authors:** Nishith R. Reddy, Hasna Maachi, Yini Xiao, Milos S. Simic, Wei Yu, Yurie Tonai, Daniela A. Cabanillas, Ella Serrano-Wu, Philip T. Pauerstein, Whitney Tamaki, Greg M. Allen, Audrey V. Parent, Matthias Hebrok, Wendell A. Lim

**Affiliations:** 1 UCSF Cell Design Institute, University of California, San Francisco, San Francisco, CA, USA; 2 Department of Cellular and Molecular Pharmacology, University of California, San Francisco, San Francisco, CA, USA; 3 Diabetes Center, University of California, San Francisco, San Francisco, CA, USA; 4 Department of Pediatrics, University of California, San Francisco, San Francisco, CA, USA; 5 UCSF CoLabs, University of California, San Francisco, San Francisco, CA, USA; 6 Parker Institute for Cancer Immunotherapy, University of California, San Francisco, San Francisco, CA, USA; 7 Helen Diller Family Comprehensive Cancer Center, University of California, San Francisco, San Francisco, CA, USA; 8 Department of Medicine, University of California San Francisco, San Francisco, CA, USA

## Abstract

Immune homeostasis requires a balance of inflammatory and suppressive activities. To design cells potentially useful for local immune suppression, we engineered conventional CD4^+^ T cells with synthetic Notch (synNotch) receptors driving antigen-triggered production of anti-inflammatory payloads. Screening a diverse library of suppression programs, we observed the strongest suppression of cytotoxic T cell attack by the production of both anti-inflammatory factors (interleukin-10, transforming growth factor–β1, programmed death ligand 1) and sinks for proinflammatory cytokines (interleukin-2 receptor subunit CD25). Engineered cells with bespoke regulatory programs protected tissues from immune attack without systemic suppression. Synthetic suppressor T cells protected transplanted beta cell organoids from cytotoxic T cells. They also protected specific tissues from unwanted chimeric antigen receptor (CAR) T cell cross-reaction. Synthetic suppressor T cells are a customizable platform to potentially treat autoimmune diseases, organ rejection, and CAR T cell toxicities with spatial precision.

Immune homeostasis requires an intricate spatiotemporal interplay between inflammatory and tolerogenic cellular activities. Cytotoxic and inflammatory activity of engineered cells has been harnessed to treat disease, as exemplified by the success of chimeric antigen receptor (CAR) T cells directed against cancer (1, 2). Conversely, immune suppressor cells capable of locally targeted suppression would have the potential to control improper immune activation and reestablish homeostasis. Engineered immune suppressor cells could in principle remodel immune microenvironments in diverse inflammatory or autoimmune disorders and could prevent transplant rejection. Moreover, if these cells acted in a locally targeted manner, they could potentially bypass the severe and chronic toxicities associated with systemic immunosuppression.

To make effective targeted immune suppressor cells, one might redirect endogenous suppressor cells, such as regulatory T (T_reg_) cells or myeloid suppressor cells (3-7). However, we took a synthetic reconstitution approach of engineering conventional CD4^+^ T cells to make them function as localized suppressor cells. This allowed us to explore the fundamental principles and requirements for local suppression (8-10). It also allowed us to use a cell platform (conventional T cells) that is stable, well characterized, and facile to engineer.

Native immune cells launch programs that produce sets of molecular factors that, together, are often far more powerful than any individual agent. With the tools of synthetic biology, we have the capability to create bespoke multiagent response programs. Thus, we can search for non-native, alternative response programs that show synergistic suppression activity. We engineered synthetic suppressor T cells with synthetic Notch (synNotch) regulatory circuits that induce production of immunosuppressive factors. SynNotch receptors are highly programmable chimeric receptors that, upon recognition of a target antigen, induce transcription of a custom transgene payload (11-13). Because synNotch engineered T cells require an antigen to trigger production of suppressive payloads, they can act in a locally targeted manner only where such antigen is expressed.

Our engineered synthetic suppressor T cells proved effective at locally inhibiting strong cytotoxic T cell responses, such as those induced by CAR T cells. Our optimal synthetic suppressor cells acted as both sinks for proinflammatory cytokines and as sources of anti-inflammatory cytokines, mimicking the overall evolutionary design of regulatory T cells. Because synthetic suppressor T cells acted in a local manner, they protected specific tissues from unwanted CAR T cross-reaction, without compromising the effectiveness of tumor killing. We also demonstrated protection of a transplanted organ from cytotoxic T cell attack in a model of pancreatic islet transplantation. These results define the minimal requirements for effective local immune suppression and demonstrate the potential of synthetic suppressor T cells as a therapeutic platform treating autoimmunity, preventing transplant rejection, or preventing CAR T cell toxicity.

## Engineering T cells that induce custom immunosuppressive programs

To design T cells in which antigen induces production of suppressive payloads, we used a synNotch receptor to induce transcription of custom transgene payloads. We built a prototype circuit in primary human CD4^+^ T cells in which a synNotch receptor induced the production of custom immunosuppressive payloads upon recognition of a model antigen, CD19. We engineered CD4^+^ T cells—synthetic suppressor T cells—that each induced production of a single agent from a diverse library of suppressive payloads including suppressive cytokines [e.g., interleukin-10 (IL-10), IL-35, active transforming growth factor–β1 (TGFβ1)] (14), inflammatory cytokine sinks [e.g., IL-2 receptor subunit CD25, soluble tumor necrosis factor a receptor (sTNFαR)] (15), inhibitory receptors or ligands [e.g., programmed death ligand 1 (PD-L1), cytotoxic T-lymphocyte associated protein 4 (CTLA4), CD39] (16), or proliferative cytokines (e.g., IL-2) (17). CD4^+^ T cells with synNotch induction circuits produced amounts of CD25, IL-10, and TGFβ1 payloads comparable to those produced by stimulated FoxP3^+^ polyclonal T_reg_ cells in vitro (fig. S1A).

**Fig. 1.**
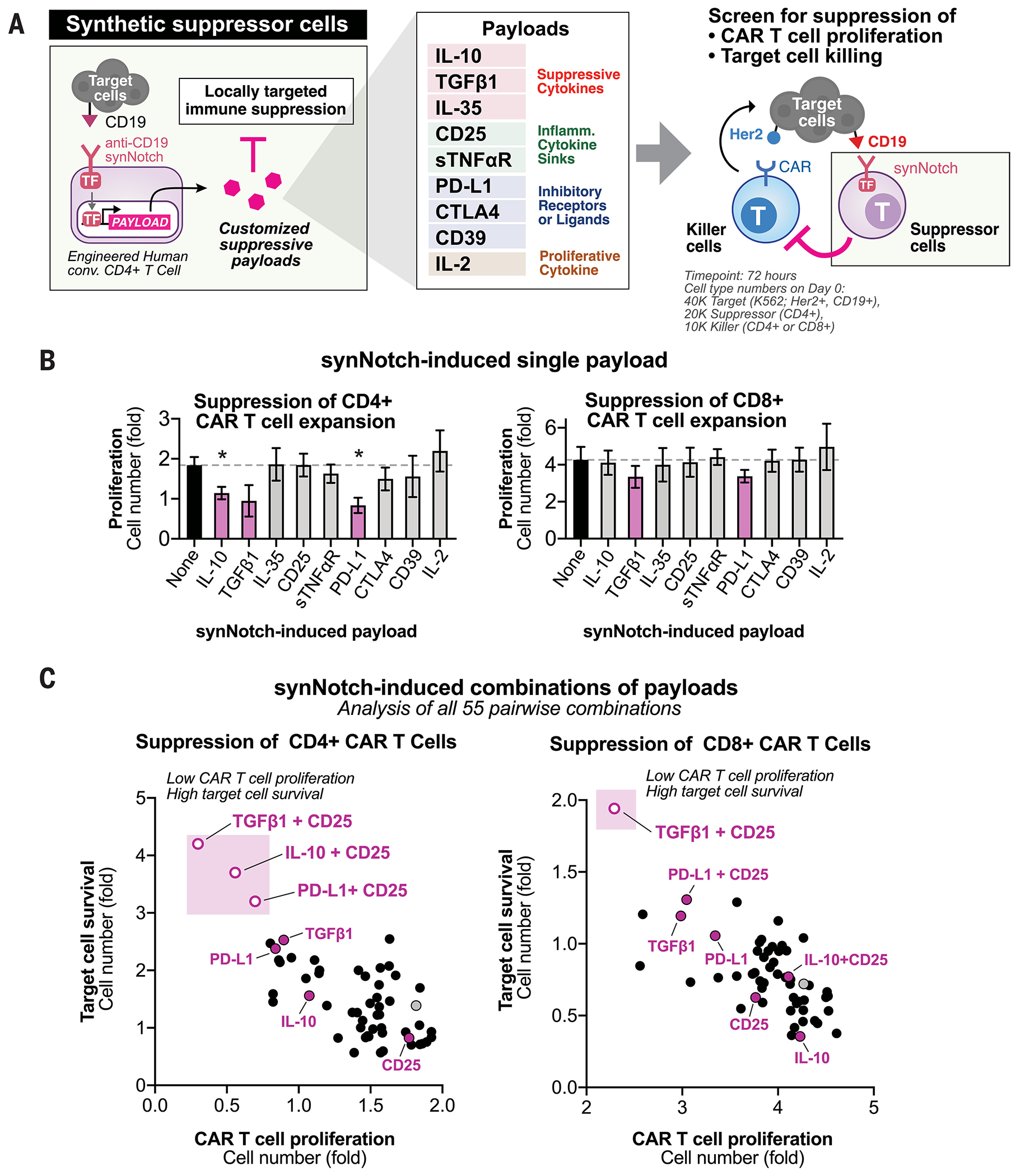
Engineering synthetic suppressor T cells that drive antigen-induced production of immune suppressive payloads. (**A**) Design of synthetic suppressor T cells that inducibly produce anti-inflammatory payloads. These are human conventional CD4^+^ T cells engineered to express a synNotch receptor that triggers the expression of a custom suppressive payload upon target antigen binding. Three-cell coculture was used to assess the ability of engineered suppressor cells to block CAR T cell proliferation and target cell killing in vitro. TF, transcription factor. (**B**) Synthetic suppressor T cells reduced proliferation of CAR T cells in vitro. As described in (A), fold proliferation of CD4^+^ and CD8^+^ CAR T cell over 72 hours in the presence of synthetic suppressor T cells with synNotch-induced individual payloads is shown. Fold change normalized to the 0 hour time point (*n* = 3 replicates, error bars = standard error). Dashed line indicates no payload suppressor T cell control. Statistical significance was tested using a two-tailed Student’s *t* test comparing to no suppressor T cell control (*P < 0.05). (**C**) Combinations of synNotch-induced payloads drove stronger suppression of CAR T cells in vitro. The fold proliferation of K562 target cells (Her2^+^, CD19^+^) and CAR T cells over 72 hours is shown for both cocultures of suppressor T cells and target cells with CD4^+^ or CD8^+^ CAR T cells. Each point indicates a pairwise combination of payloads from the library in (A) induced by anti-CD19 synNotch suppressor cells (mean, *n* = 3 replicates). Fold change normalized to the 0 hour time point. Gray point indicates the no-payload suppressor T cell control. See fig. S2, A and B, for data in (C) as a heatmap of combinatorial payloads. See fig. S3, A and B, for similar analysis with polyclonal T cells stimulated through their endogenous TCR.

We focused on identifying suppressive programs that blocked CAR T cell–mediated cytotoxicity. Many autoimmune disorders and rejection phenomenon are driven by pathogenic T cells. There are few suppressive therapies that effectively block T cells, in contrast to the many effective suppressive therapies that block B cell activity (e.g., antibody to CD20 or anti-CD19 CAR T cells) (6, 18). To identify minimal modules that suppressed T cell–mediated killing, we assessed the effectiveness of synthetic suppressor T cells in blocking the strong cytotoxicity induced by CD4^+^ and CD8^+^ CAR T cell activity in vitro where each suppressor cell produced a single agent from a library of payloads (Fig. 1A). In a three-cell suppression assay, we cultured together (i) synthetic suppressor cells; (ii) anti-Her2 CAR T cells (19); and (iii) target cells (K562, Her2^+^, CD19^+^) that express both the CAR cognate antigen (Her2) and the synNotch cognate antigen (CD19). We measured suppression by tracking survival of target cells as well as inhibition of CAR T cell proliferation. Suppressor cells with inducible production of TGFβ1, PD-L1, or IL-10 suppressed the proliferation of CD4^+^ CAR T cells, whereas inducible production of TGFβ1 or PD-L1 (but not IL-10) suppressed the proliferation of CD8^+^ CAR T cells in vitro (Fig. 1B and fig. S1B). The remaining synNotch-induced single payloads tested did not significantly inhibit proliferation of CAR T cells. This demonstrates that suppression of T cell activity using specific single anti-inflammatory payloads is feasible and exhibits some specificity for CD4^+^ or CD8^+^ T cell activity.

Synthetic suppressor T cells are not subject to self-inactivation. The custom suppressive programs depend on synNotch activation and not on T cell receptor (TCR) or CAR activation—the pathways that are inhibited in immune suppression. In the in vitro suppression assay described above, the suppressor T cells maintained activation of synNotch circuits (measured by induction of an mCherry reporter), even in the presence of immunosuppressive factors such as TGFβ1 because synNotch bypasses the requirements for TCR signaling (fig. S1C). The orthogonality of synthetic suppression programs allowed them to remain stable despite producing payloads that inhibit TCR signaling.

## Combination payloads synergistically suppress cytotoxic T cells

Natural immunoregulatory cells act through multiple pathways to suppress immune activation (20, 21). We therefore tested whether combined immunosuppressive payloads might improve inhibition of cytotoxic T cell activity by engineered suppressor cells. We engineered synthetic suppressor cells that conditionally produced all possible two-agent payload combinations (Fig. 1C). We engineered human primary CD4^+^ T cells by dual lentiviral transduction, in which each lentivirus introduced one synNotch-induced custom transgene, creating all 55 pairwise combinations of suppressive payloads. The ability of synthetic suppressor T cells to block the proliferation and cytotoxicity of CD4^+^ CAR T cells or CD8^+^ CAR T cells was then evaluated in vitro in suppression assay with target cells as described above.

The most effective programs for suppression always included induction of an inhibitor of T cell activation (TGFβ1, IL-10, or PD-L1) and a sink for IL-2 (CD25) (Fig. 1C and fig. S2). No other combination of the tested suppressive factors within the same class or between classes showed significant synergistic benefit. TGFβ1 was an effective inhibitor of both CD4^+^ and CD8^+^ T cell cytotoxicity, whereas IL-10 was only active against CD4^+^ T cells. Synthetic payload combinations with PD-L1 (a feature of myeloid suppressor cells) (4) and CD25 (a feature of T_reg_ cells) (21) also drove effective suppression.

We also tested the combinatorial library of suppressor programs against polyclonal human primary CD4^+^ and CD8^+^ T cells activated through their endogenous TCR by anti-CD3/CD28 antibodies in vitro (rather than CAR T cells). We observed that a similar set of signals—specifically combinations of an inhibitory payload (TGFβ1, IL-10, PD-L1, sTNFαR) with CD25—could effectively suppress proliferation of TCR-stimulated polyclonal CD4 and CD8 T cells [measured by cell counts and carboxyfluorescein diacetate succinimidyl ester (CFSE) CellTrace dilution], analogous to what we observed against CAR T cells (fig. S3). These results suggest that these suppressor programs could potentially be applied to inhibit TCR activation in addition to CAR activation.

**Fig. 2.**
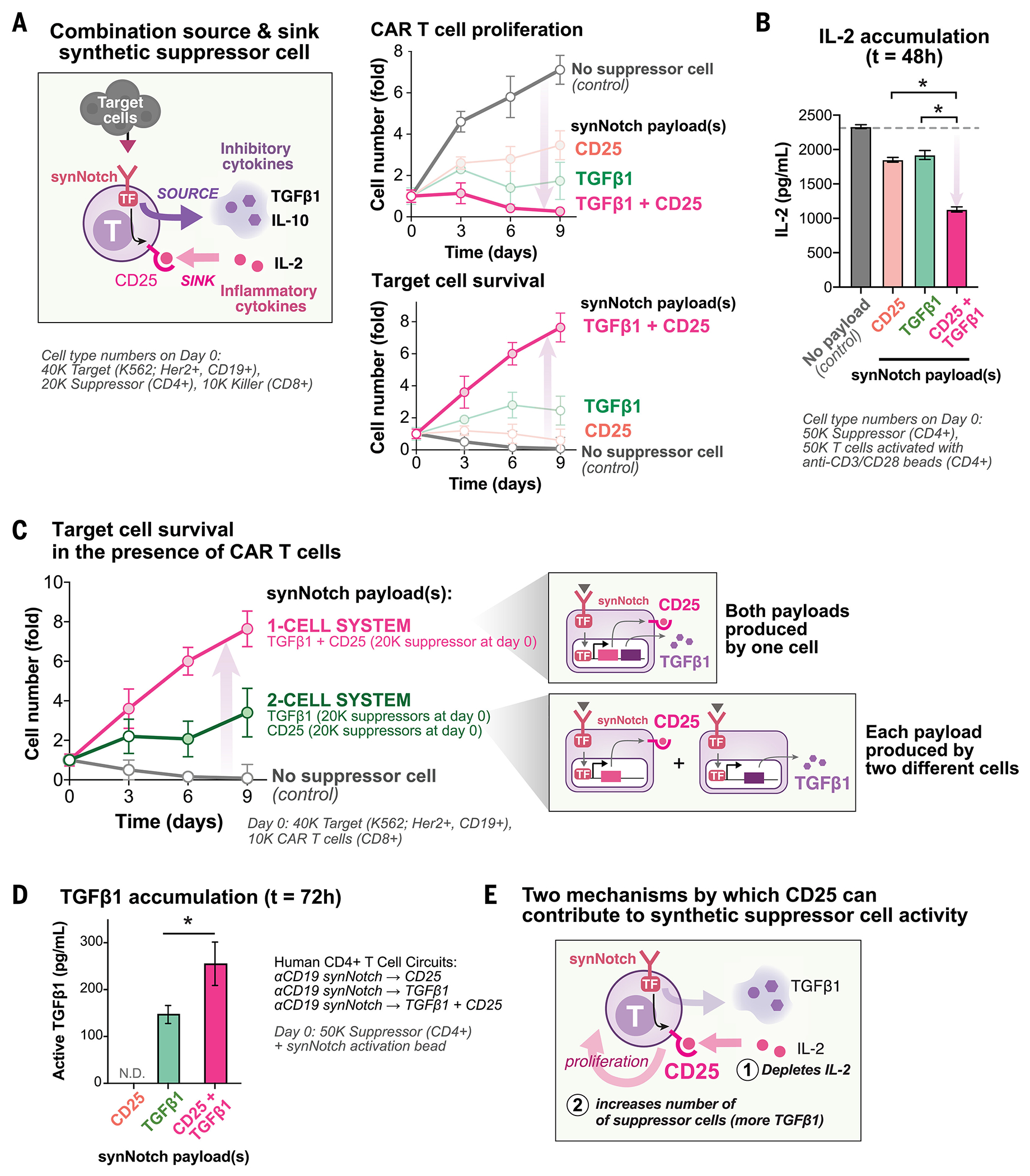
Combinatorial induction of both CD25 and TGFβ1 by the same suppressor cell leads to more effective suppression of CAR T cells in vitro. (**A**) Synthetic suppressor T cells that act as a source for inhibitory cytokines and a sink for inflammatory cytokines drove stronger suppression of CAR T cells in vitro. Synthetic suppressor T cells that induced a combination of TGFβ1 and CD25 were more potent at suppressing CD8^+^ CAR T cell activity compared with each individual payload alone. Cell counts are normalized to the 0 hour time point (*n* = 3 replicates, error bars = standard error, filled markers indicate two-tailed *t* test, *P* < 0.05, comparison to no-suppressor cell control). (**B**) Synthetic suppressor T cells depleted IL-2 produced by activated CD4^+^ T cells in vitro. Human CD4^+^ T cells activated by anti-CD3/CD28 beads for 24 hours were cocultured with synthetic suppressor T cells activated with synNotch activating beads (anti-Myc beads). The IL-2 levels in the supernatant were measured by ELISA (*t* = 48 hours, *n* = 3 replicates, error bars = standard error, two-tailed *t* test comparing TGFβ1 and CD25 to each payload alone, *P < 0.05). (**C**) Synthetic suppressor T cells required both TGFβ1 and CD25 to be produced by the same cell for effective suppression in vitro. Separation of TGFβ1 and CD25 into two separate cells led to weaker suppression of CD8^+^ CAR T cell killing (reduced target-cell proliferation) than a one-cell system where both payloads are produced by the same suppressor T cell in vitro (*n* = 3 replicates, error bars = standard error, filled markers indicate two-tailed *t* test, *P* < 0.05, comparison to no-suppressor cell control). (**D**) CD25 drives increased TGFβ1 production by synthetic suppressor T cells in vitro. Suppressor cells that induced a combination of TGFβ1 and CD25 led to more TGFβ1 accumulation than suppressor cells inducing TGFβ1 alone. Suppressor cells were activated in vitro with synNotch activation beads (anti-Myc beads). TGFβ1 levels were measured by ELISA of supernatant (*t* = 72 hours, *n* = 3 replicates, error bars = standard error, two-tailed *t* test between TGF β1 circuit with and without CD25, *P < 0.05). (**E**) CD25 can enhance suppressor cell activity by two mechanisms. CD25 depletes IL-2 from the local microenvironment and drives preferential proliferation of suppressor cells. An increase in suppressor cell number can yield higher TGFβ1 accumulation.

The most effective suppression of T cell cytotoxicity across both CD4^+^ and CD8^+^ T cells was observed with cells that produced both TGFβ1 and CD25 (Fig. 1C). This combination payload showed strong synergy, outperforming each individual payload at suppressing CD8^+^ CAR T cell proliferation and cytotoxicity in vitro (Fig. 2A). We tested a dose titration of suppressor cells in vitro and assessed suppression of cocultured CD8^+^ CAR T cells. Circuits that induced the combination of TGFβ1 and CD25 increased both the maximum amplitude (maximal level of suppression) and reduced the EC_50_ (suppressor cell dose required for 50% maximal response) for suppression of CD8^+^ CAR T cells compared with induction of each payload alone—as measured by CAR T cell proliferation and target cell protection (fig. S4A). Local accumulation of IL-2 is a critical requirement for the proliferation of cytotoxic T cells (13). Suppressor cells with a synNotch→TGFβ1+CD25 circuit reduced the accumulation of IL-2 produced by activated CD4^+^ T cells more effectively than suppressor cells that produced each payload alone in vitro (Fig. 2B and fig. S4B). Similarly, suppressor T cell circuits with simultaneous production of both IL-10 and CD25 or TGFβ1 and CD25 were effective at suppressing the proliferation and cytotoxicity of CD4^+^ CAR T cells (fig. S4C).

To evaluate the mechanism of synergy between the anti-inflammatory payloads and the inflammatory cytokine sink payloads, we reconfigured these circuits such that each payload was produced by a separate suppressor cell population (one suppressor cell produced CD25, the IL-2 sink, and another cell produced the inhibitory cytokine TGFβ1). The two-cell system was considerably less effective at CAR T cell suppression as observed by inhibition of CAR T proliferation and cytotoxicity (Fig. 2C). Thus, synNotch-induced production of CD25 and an inhibitory cytokine appears best produced by the same suppressor T cell for effective suppression.

When overexpressed in CD4^+^ T cells, we reasoned that CD25 can act both as a sink for IL-2 and as a way to drive preferential proliferation of the engineered T cells expressing CD25 (fig. S5A) (CD25 is a subunit of the high-affinity IL-2 receptor complex). Suppressor T cells with a synNotch→TGFβ1+CD25 circuit exhibited stronger IL-2 receptor signaling with increased abundance of CD25 and phosphorylated STAT5 (signal transducer and activator of transcription 5) levels compared with those in cocultured CD4^+^ or CD8^+^ CAR T cells during suppression in vitro (fig. S5B). We also observed that synthetic suppressor T cells expressing CD25 show strong preferential proliferation compared with cocultured CD8^+^ CAR T cells during suppression in vitro (fig. S5C). The co-induction of CD25 by suppressor T cells yielded higher concentrations of TGFβ1 in the medium than did circuits that induced expression of TGFβ1 alone in vitro (Fig. 2D). Thus, CD25 appears to contribute to enhanced suppression by two mechanisms, both enhancing the consumption of IL-2 and driving preferential proliferation of suppressor cells, which creates a positive feedback loop to further increase local TGFβ1 production (Fig. 2E).

To dissect the mechanism of CAR T cell inactivation by suppressor T cells in greater detail, we analyzed CAR T cell activation states and cytokine secretion during suppression in vitro. Suppressor T cells with a synNotch→TGFβ1+CD25 circuit effectively decreased accumulation in the media of proinflammatory cytokine interferon-γ (IFN-γ) produced by activated CD4^+^ CAR T cells in vitro (fig. S6A). We performed intracellular staining of CAR T cells in suppression coculture to assess the effects of suppression at a single-cell level. We stained CAR T cell markers of degranulation (granzyme B), cytokine production (IL-2, IFN-γ, TNFα), and proliferation (Ki67). For both CD4^+^ and CD8^+^ CAR T cells, we observed the most reduction in CAR T cell proliferation (decreased Ki67) and degranulation (decreased granzyme B) with suppression, but we also observed reduced cytokine production in individual CAR T cells (fig. S6B). These results suggest that suppressor circuits inhibit CAR T cells by both affecting T cell proliferation and impairing cytotoxic activities (degranulation and cytokine production).

Overall, these engineered circuits share a common design for effective suppressor T cells (against cytotoxic T cells); they produce a source (the inhibitory cytokine) and a cytokine sink (the high-affinity IL-2 receptor CD25). Natural T_reg_ cells share these characteristics.

## Synthetic suppressor cells locally inhibit T cell killing without systemic suppression in vivo

Most treatments for inflammatory disorders also cause systemic immunosuppression. The risks associated with long-term systemic immunosuppression present a major barrier for these interventions. Cell-based therapies with precise molecular recognition could in principle be used to target immune suppression locally without affecting immunity in off-target areas. We therefore tested local suppression of immune responses by synthetic suppressor cells in a two-tumor model in vivo.

**Fig. 3.**
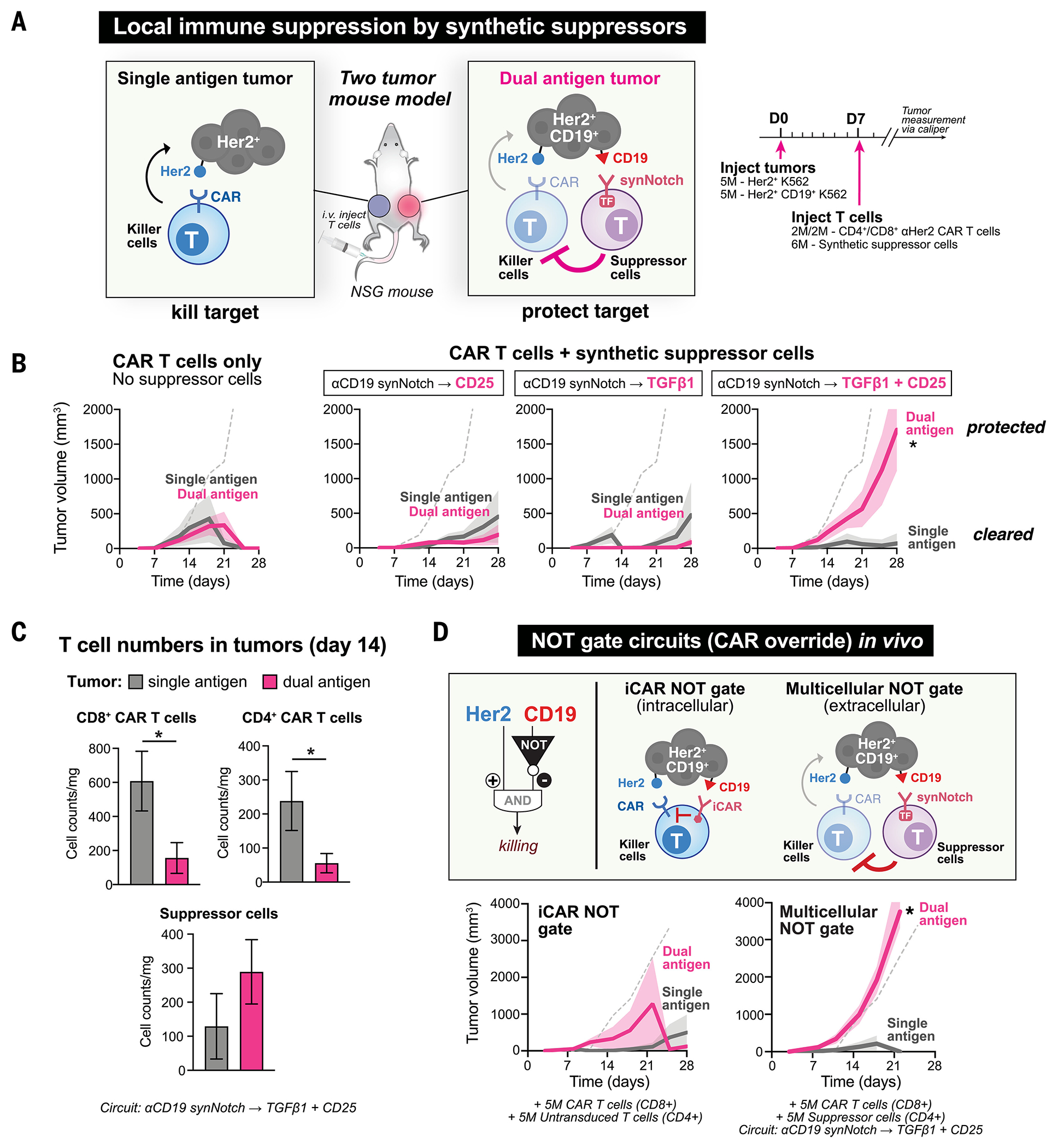
Synthetic suppressor cells block CAR T cell killing in vivo in locally targeted manner. (**A**) Two-tumor mouse model was used to assess local immune suppression. Two tumors were injected subcutaneously into immunocompromised NSG mice, such that the right flank had a dual-antigen tumor (Her2^+^ CD19^+^ K562 tumor) and the left flank had a single-antigen tumor (Her2^+^ K562 tumor). Anti-Her2 CAR T cells and anti-CD19 synNotch suppressor T cells were injected intravenously. Tumor volumes were measured by calipers. (**B**) Synthetic suppressor T cells can block CAR T cell killing locally without systemic suppression. Suppressor T cells (anti-CD19 synNotch→TGFβ1+CD25) are effective at blocking CAR T cell killing of the dual-antigen tumor (CD19^+^) without compromising killing of the single antigen tumor (CD19^−^). Suppressor cells producing each payload alone were not sufficient to protect the dual-antigen tumor from CAR T cell killing. Tumor measurements shown as time after T cell injection (*n* = 5 replicates, solid line = mean, shading = standard error, two-tailed *t* test, *P < 0.001 on day 28). Dashed gray line indicates tumor growth with no T cell injection. See fig. S7 for tumor growth curves for individual mice. (**C**) Synthetic suppressor T cells reduced CAR T cell proliferation in dual-antigen tumor in vivo. Flow profiling of isolated tumors at day 14 showed reduced accumulation of both CD4^+^ and CD8^+^ CAR T cells (GFP^+^) and an increased accumulation of suppressor cells (BFP^+^) in the dual-antigen tumor. Cell counts normalized to tumor weight after isolation (*n* = 3 replicates, error bars = standard error, two-tailed *t* test, *P < 0.05). (**D**) Multicellular NOT gate tumor-killing circuit combining CAR T cells and synthetic suppressor T cells drove robust local suppression. Multicellular NOT gate circuit leads to more-robust local suppression than iCAR NOT circuit in two-tumor model in vivo (25). iCAR NOT gate circuit (anti-Her2 CAR + anti-CD19 PD-1 iCAR) fails to block killing of the dual-antigen tumor. In the multicellular NOT gate tumor-killing circuit, anti-Her2 CAR T cells recognize and kill both tumors, whereas anti-CD19 synthetic suppressor T cells block killing in the CD19^+^ dual-antigen tumor (*n* = 5 replicates, solid line = mean, shading = standard error, two-tailed *t* test, *P < 0.001 day 21). Dashed gray line indicates tumor growth with no T cell injection. Additional replicates shown in fig. S9A, and tumor growth curves for individual mice shown in fig. S9B.

We implanted K562 tumors (a human leukemia cell line) subcutaneously into two flanks of immunocompromised nonobese diabetic *scid* gamma (NSG) mice. Both tumors were modified to express the antigen Her2, which can be targeted by anti-Her2 CAR T cells to kill the tumors. Only one tumor, however, also expressed CD19, the antigen that activated the synNotch receptor in the engineered suppressor T cells (Fig. 3A). Our goal was to test whether the synthetic suppressor cells could locally protect the CD19^+^ tumor from CAR T killing but leave the CD19^−^ tumor still subject to efficient CAR T killing.

CAR T cells injected intravenously without suppressor cells cleared both tumors equally well (Fig. 3B). However, when synthetic suppressor T cells with the synNotch→TGFβ1+CD25 circuit were injected along with CAR T cells, CAR T cell killing of the dual-antigen tumor (Her2^+^, CD19^+^) was suppressed, without affecting clearance of the single-antigen tumor (Her2^+^, CD19^−^) (Fig. 3B and fig. S9). Suppressor T cells that induced the individual payloads TGFβ1 or CD25 alone failed to protect the dual-antigen tumor from CAR T cell killing in this model, whereas suppressor cells that expressed both payloads showed good protection. We isolated both tumors at day 14 and analyzed T cell counts by flow cytometry, separating CAR T cells [green fluorescent protein (GFP)–labeled] and suppressor T cells [blue fluorescent protein (BFP)–labeled]. Treatment with suppressor cells with the synNotch→TGFβ1+CD25 circuit reduced accumulation of both anti-Her2 CD4^+^ and CD8^+^ CAR T cells in the dual-antigen tumor at day 14 (Fig. 3C). Conversely, we observed increased accumulation of synthetic suppressor T cells in the dual-antigen tumor (synNotch antigen CD19^+^ cells).

We compared the local inhibition of CAR T cells by synthetic suppressor T cells to isolated human FoxP3^+^ polyclonal T_reg_ cells or FoxP3^+^ T_reg_ cells engineered with an anti-CD19 CAR in vitro and in vivo. We observed comparable suppression of CD8^+^ CAR T by polyclonal T_reg_ cells (prestimulated for 24 hours using anti-CD3/CD28 antibody) compared with synthetic suppressor T cells with the synNotch→TGFβ1+CD25 circuit in vitro (fig. S8, A and B). Anti-CD19 CAR T_reg_ cells (with CD28 costimulatory domain), however, failed to show significant suppression in vitro (fig. S8C). Both polyclonal and CAR-engineered T_reg_ cells failed to show local suppression of CAR T cell killing of the dual-antigen tumor in vivo in the two-tumor mouse model (fig. S8D) and therefore, in this assay, performed less effectively than the synthetic suppressor cells. Thus, the synthetic suppressor T cells could exhibit strong and effective local suppression of CAR T cells but did not induce systemic suppression (CAR T cells can still kill targets not colocalized with the suppressor-inducing antigen).

## Protecting cross-reactive tissues from CAR T killing in vivo

A locally acting suppressor cell could be useful to block killing of nontumor tissue by CAR T cells in cases where a CAR T cell cross-reacts with healthy tissue. Such nontumor cross-reactivity of CAR T cells remains a toxicity challenge to the application of CAR T cells to solid tumors, which often lack absolutely tumor-specific target antigens (22, 23). To address this, Boolean logic gates using synthetic molecular switches have been introduced into CAR T cells to increase their specificity and limit off-target toxicity (24). Inhibitory CARs (iCARs) have been used as a Boolean NOT gate that can block CAR T cell killing in an antigen-dependent manner—the iCAR recognizes an overriding “NOT” antigen only expressed on the normal tissue, activating an inhibitory intracellular response (from PD1 or CTLA4) that can block CAR activation. Efficacy of iCARs depends on the abundance of target antigen and binding affinity of the extracellular recognition domain (25). However, in our two-tumor mouse model, CD8^+^ T cells expressing both an anti-Her2 CAR and anti-CD19 iCAR failed to effectively block CAR T cell killing of the dual-antigen (Her2^+^, CD19^+^) tumor (Fig. 3D).

The synthetic suppressor cell described here can serve as an alternative NOT gate when combined with a CAR T cell. We treated a two-tumor mouse model with anti-Her2 CAR T cells (CD8^+^) along with a suppressor T cell (CD4^+^) expressing an anti-CD19 synNotch→TGFβ1+CD25 circuit. This multicellular system led to consistent protection of the dual-antigen (Her2^+^, CD19^+^) tumor without blocking CAR T cell killing of the single-antigen (Her2^+^) tumor. Local suppression of CAR T cell killing in the dual-antigen tumor without compromising killing of the single-antigen tumor was highly reproducible in T cells from three independent human T cell donors (fig. S9, A and B). These experiments demonstrate that suppressor cells can be programmed to protect cross-reactive normal tissues or organs defined by a specific NOT antigen without affecting on-target tumor killing. By minimizing the risk of off-target or on-target toxicity to nontumor cells, synthetic suppressor T cell circuits could expand the repertoire of possible tumor antigens to target (22).

## Synthetic suppressor cells protect local bystander cells

Synthetic suppressor cells with the synNotch→TGFβ1+CD25 circuit are thought to use a paracrine signaling mechanism to drive suppression. Therefore, we tested whether these circuits could protect bystander target cells (i.e., neighboring cells that express the killing target antigen, Her2, but not the synNotch antigen, CD19) when colocalized with CD19^+^ cells that trigger the suppressive program. In short, suppression should be able to overcome the heterogeneous expression of synNotch priming antigen with target cells through paracrine action in the local immune microenvironment. Thus, to assess bystander cell protection, we mixed dual-antigen (Her2^+^, CD19^+^) target cells and single-antigen (Her2^+^) bystander target cells in vitro at a 1:1 ratio. Both types of cells can be CAR T killing targets, but only the CD19^+^ cells can serve to induce the suppressive response. Bystander target cells (synNotch antigen CD19^−^ cells) were labeled with GFP, allowing for discrimination of the distinct target cell populations by flow cytometry. When this target cell mixture was cultured with CAR T cells and suppressor T cells, both types of target cells proliferated in vitro at similar rates. Thus, these circuits appear to protect bystander target cells that lacked the synNotch antigen (fig. S10A).

In the two-tumor NSG mouse model, we also assessed suppression with a heterogeneous tumor (varied percentage of dual-antigen target cells) by mixing different ratios of dual-antigen and single-antigen target cells. All mice were also injected subcutaneously with a single-antigen tumor that lacked a synNotch priming antigen in the alternate flank. This single-antigen tumor, which completely lacked any synNotch priming antigen, was cleared by CAR T cells in vivo in all cases. We observed effective local suppression of CAR T cell killing of the tumor containing only 25% dual-antigen target cells at the time of engraftment, while still observing clearance of the single-antigen tumors in the opposing flank (fig. S10B). Thus, synthetic suppressor cells could still function effectively in the presence of heterogeneity in synNotch antigen expression. The suppressor cells should protect target cells in the local region of the inducing antigen but not cause systemic immune suppression.

## Synthetic suppressor cells inhibit T cell killing of islet-like organoids

Another case in which local immune suppression would be desirable is in allogeneic transplantation of solid organs, which is limited by host immune rejection and requires long-term systemic immunosuppression to protect grafts (26, 27). We assessed whether engineered suppressor T cells could protect transplanted organs from immune rejection in a model of pancreatic islet transplantation. Transplantation of primary human islets or human pluripotent stem cell (hPSC)–derived islet cells to replace dysfunctional or damaged pancreatic islets is a promising therapy to treat type 1 diabetes. However, like solid organ transplantations, these therapies often fail owing to host immune rejection of the transplant and because of direct effects of commonly used immunosuppressive medications on islet survival and function (28-30).

**Fig. 4.**
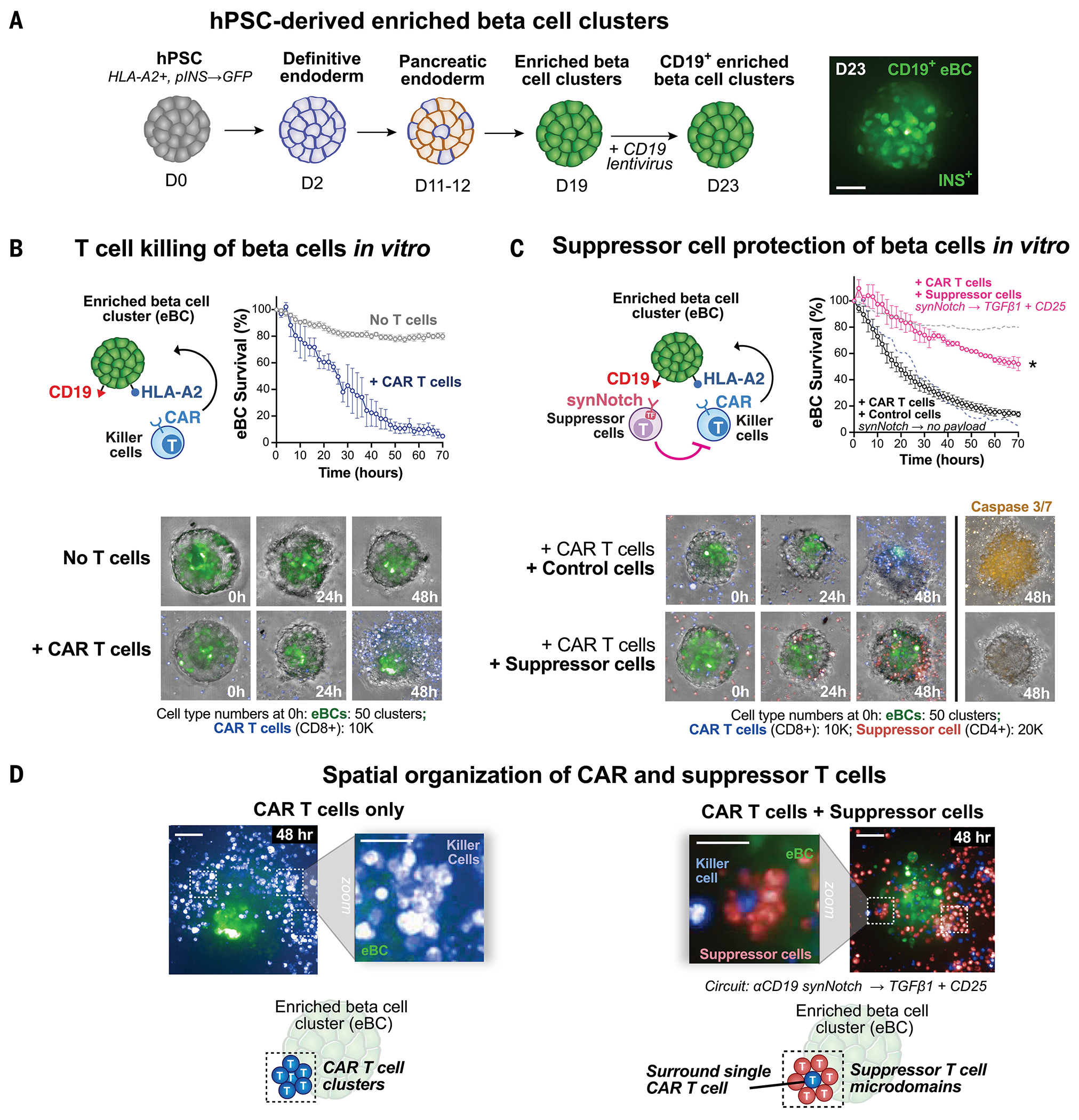
Synthetic suppressor cells protect beta cells from T cell–mediated destruction in vitro. (**A**) eBC organoids were generated from hPSCs. eBC organoids were differentiated from hPSCs as previously described (31). eBC organoids were engineered to express model antigen CD19 by lentiviral transduction on day 19 of differentiation. eBC organoids were HLA-A2^+^ and expressed GFP under the control of the insulin promoter. Confocal microscopy (maximum projection) of an eBC is shown on day 23 of differentiation. Coculture with T cells was performed on day 26 of differentiation. Scale bar, 100 μm. (**B**) Cytotoxic T cells can kill eBC organoids. Human anti-HLA-A2 CAR CD8^+^ T cells cocultured with HLA-A2^+^ eBC organoids effectively killed eBC organoids in vitro. Confocal microscopy (maximum projection) showed eBC organoid destruction mediated by CAR T cells in vitro after 48 hours (*n* = 3 replicates, error bars = standard error). (**C**) Synthetic suppressor T cells protected beta cells from cytotoxic T cell killing. eBC organoids were cocultured with T cells as in (B). Anti-HLA-A2 CAR T cell killing of eBCs was blocked by synthetic suppressor T cells (anti-CD19 synNotch→TGFβ1+CD25 circuit) but not by no-payload control cells (anti-CD19 synNotch→mCherry). Dashed lines indicate CAR-only control (blue) and no T cell control (gray) (*n* = 3 replicates, error bars = standard error, two-tailed *t* test, *P < 0.001 at 70 hours comparing control cells to suppressor cells). Confocal microscopy (maximum projection) shows protection of an eBC organoid with suppressor T cells. Caspase 3/7 dye was used to label apoptotic cells and imaged (maximum projection) at the 48-hour time point. (**D**) Synthetic suppressor T cells self-organized around cytotoxic T cells during suppression in vitro. Suppressor T cells spatially self-organized around individual activated CAR T cells during suppression (*t* = 48 hours), blocking the formation of CAR T cell clustering that is normally observed in target killing in the absence of suppression. Scale bars, 100 μm (zoomed-out image) and 25 μm (zoomed-in image).

We tested whether synthetic suppressor T cells could protect islet-like organoids from cytotoxic T cell killing. We differentiated enriched beta cell (eBC) organoids from hPSCs (31) and engineered them to express a model antigen, CD19 (Fig. 4A). eBC organoids express GFP under the control of the insulin promoter and are HLA-A2^+^ (HLA-A2, human leukocyte antigen-A2; fig. S11A) (32). Cytotoxic T cell killing of these islet-like organoids can be modeled using CD8^+^ T cells expressing an anti–HLA-A2 CAR (33-35). Anti–HLA-A2 CAR T cells kill eBC organoids in vitro within 72 hours (Fig. 4B).

We tested whether suppressor cells expressing an anti-CD19 synNotch could block anti–HLA-A2 CAR T cells from killing the target eBCs. The synNotch-activated suppressor cells were engineered to express an mCherry reporter when activated by CD19^+^ eBC organoids (fig. S11B), in addition to the TGFβ1+CD25 combinatorial payload. The suppressor cells protected CD19^+^ eBC organoids from anti–HLA-A2 CAR T cell killing (and reduced apoptosis, as measured by caspase 3 or 7 signal). In contrast, a control synNotch circuit inducing no payload failed to block cytotoxicity against eBCs (Fig. 4C).

Suppressor T cells with synNotch→TGFβ1+CD25 circuits self-organized around the attacking CAR T cells during suppression in vitro, preventing the formation of large CAR T clusters that formed in their absence. Single CAR T cells in contact with the eBCs were surrounded by synthetic suppressor T cells after ~48 hours, forming microdomains (Fig. 4D). Spatial organization observed with these minimal components resembled the close interactions between regulatory T cells and effector T cells in lymph nodes (36, 37). This organization of suppressor cells may limit transmission of proinflammatory signals between effector T cells, such as IL-2, which is required to mount a strong immune response.

## Synthetic suppressor cells protect islet organoid transplants from T cell killing in vivo

**Fig. 5.**
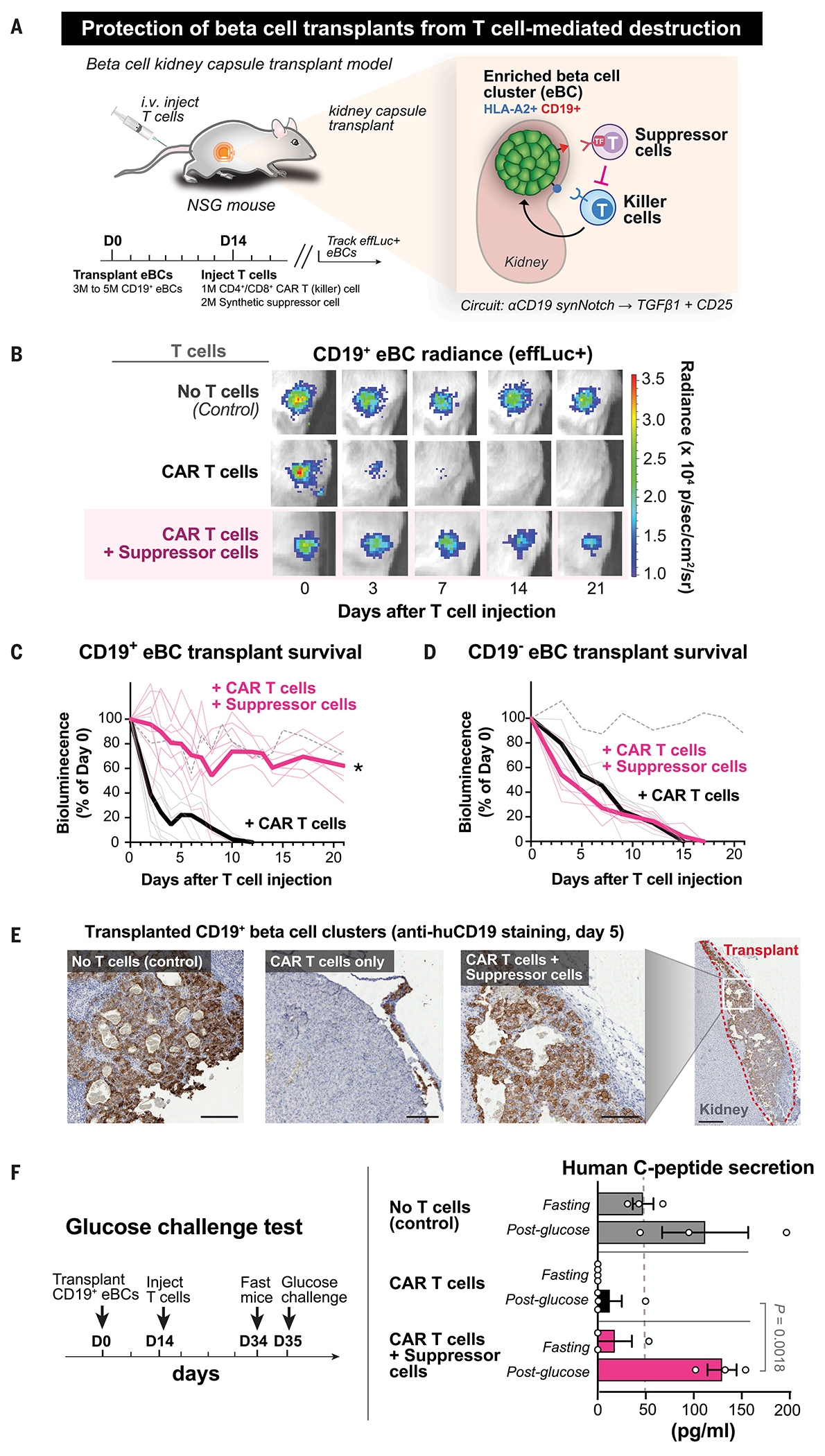
Synthetic suppressor cells locally protect hPSC-derived beta cell transplants from T cell–mediated killing in vivo. (**A**) Transplant rejection was modeled by cytotoxic T cell rejection of transplanted eBC organoids under the kidney capsule of immunocompromised NSG mice. Fourteen days after transplantation, T cells were coinjected intravenously. eBC organoids express luciferase, allowing for noninvasive imaging of transplant survival. (**B**) Synthetic suppressor T cells blocked cytotoxic T cell killing of eBC organoid transplants. Bioluminescence imaging was used to track eBC organoid survival. Human anti-HLA-A2 CAR T cells alone cleared transplants within 2 weeks. However, transplants remained intact when synthetic suppressor T cells (anti-CD19 synNotch→TGFβ1+CD25 circuit) were coinjected along with CAR T cells. (**C**) Synthetic suppressor T cells protect eBC organoid transplants with synNotch priming antigen (CD19^+^). Survival of CD19^+^ eBC organoid transplants as in (B) is assessed by noninvasive imaging (*n* = 6 to 8 replicates, two-tailed *t* test, *P < 0.001 comparing CAR T cell condition with and without suppressor T cells). Increased survival of eBC organoid transplants was observed with synthetic suppressor T cells (anti-CD19 synNotch→TGFβ1+CD25 circuit), but all transplants were cleared by anti-HLA-A2 CAR T cells alone. Dashed line indicates no–T cell control (*n* = 3 replicates, mean). (**D**) Synthetic suppressor T cells did not protect eBC organoid transplants that lack the synNotch priming antigen. Survival of CD19^−^ eBC organoid transplants is assessed as in (B). No survival advantage was observed in the presence or absence of suppressor T cells in all cases (*n* = 5 replicates, two-tailed *t* test, *P < 0.001 comparing CAR T cell condition with and without suppressor T cells). Dashed line indicates no–T cell control (*n* = 3 replicates, mean). (**E**) Transplanted eBC organoids (CD19^+^) maintain their structure in the presence of synthetic suppressor T cells but are cleared by CAR T cells alone. eBC organoids were transplanted as in (B). Anti-human CD19 staining was used to identify transplanted eBC organoids in isolated mouse kidneys from transplanted mice 5 days after T cell injection. Staining shows survival of transplants in no–T cell control and CAR T cell in the presence of suppressor cells. Minimal human CD19 staining was observed in the CAR T cell–only condition. Scale bars, 100 μm (zoomed-in images) and 500 μm (zoomed-out image). See fig. S12A for anti-human CD19 and insulin staining of adjacent tissue section. (**F**) Transplanted eBC organoids retain endocrine function after synthetic suppressor T cell protection. Glucose challenge test was performed on NSG mice with eBC organoid transplants 21 days after injection of T cells (35 days after transplantation). Human C-peptide during fasting conditions and 30 min after intraperitoneal glucose injection (*n* = 3 or 4 replicates, error bars = standard error) was measured by ELISA of blood serum. Glucose challenge showed that eBC organoids in mice injected with synthetic suppressor T cells remain functional and can secrete human C-peptide after glucose stimulation. *P* = 0.0018, two-tailed *t* test between CAR T cells with and without suppressor cells after glucose injection.

To evaluate the potential of synthetic suppressor T cells to protect organoid transplants from cytotoxic T cell killing in vivo, luciferase-expressing eBC organoids were transplanted under the kidney capsule of NSG mice (Fig. 5A). To model strong immune rejection of transplants by cytotoxic host T cells, anti–HLA-A2 CAR T cells (CD4^+^ and CD8^+^) were injected to drive cytotoxicity against the organoids (which are HLA-A2^+^). The constitutive expression of luciferase in these organoids allows for noninvasive imaging of the transplant. The survival of the grafts was tracked by bioluminescence in the presence or absence of suppressor cells (anti-CD19 synNotch→TGFβ1+CD25 circuit) (Fig. 5B).

Without infusion of CAR T cells, eBC organoid grafts survived and were detectable by bioluminescence for at least 6 weeks. Injection of CAR T cells alone drove clearance of the CD19^+^ eBC organoid transplant within 12 days in all cases (Fig. 5C). However, when synthetic suppressor T cells were injected intravenously along with CAR T cells, CD19^+^ eBC organoids were protected from T cell killing in vivo, with effective suppression observed in six of eight replicates. To test antigen-specific suppression of cytotoxic T cell killing by synthetic suppressor cells, experiments were conducted with transplanted eBC organoids lacking the synNotch antigen, CD19 (Fig. 5D). Suppression of T cell killing was dependent on the presence of the synNotch antigen, CD19, on the transplanted cells, and no protection of eBC organoid transplants lacking CD19 was observed in the presence of CAR T cells and suppressor cells in all cases.

To profile transplanted eBC organoids in more detail, we isolated transplants for histology and flow cytometry analysis 5 days after T cell injection. In the presence of suppressor T cells, transplants remained intact, as observed by anti-CD19 staining of isolated CD19^+^ eBC transplants (Fig. 5E). Staining of isolated CD19^+^ eBC transplant sections by multiplexed ion beam imaging (MIBI) shows that transplants maintained higher concentrations of insulin when treated with suppressor T cell injection, as compared with CAR T cells alone (fig. S12A). Flow analysis of isolated CD19^+^ eBC transplants show that CAR T cell proliferation was significantly reduced in the eBC transplant but not reduced in off-target tissue such as the spleen (fig. S12B). Suppressor T cells appear to locally block CAR T proliferation in the transplant without systemic suppression. To determine whether suppressor T cells generated high levels of TGFβ1 in circulation, we measured TGFβ1 in isolated blood, spleen, and transplant samples on day 20 after T cell injection. We observed a detectable increase in TGFβ1 in the transplant with suppressor T cells but no detectable increase in TGFβ1 in circulating blood or the spleen. Thus, suppressor T cells produce TGFβ1 locally in the transplant (fig. S12C).

To evaluate whether eBC organoid graft function was maintained with suppressor protection, we quantified glucose-stimulated insulin secretion by the transplanted organoids. At 35 days after transplantation, mice were fasted, and the amount of human insulin connecting peptide (C-peptide) levels were measured before and after (30 min) intraperitoneal administration of glucose (Fig. 5F). In the group of mice transplanted with organoids cleared by CAR T cells in the absence of suppressor cells, all but one animal showed no detectable human C-peptide secretion. In the presence of synthetic suppressor T cells, however, transplants remained functional, producing amounts of glucose-stimulated human C-peptide comparable to those observed with eBC transplants not treated with CAR T cells. Thus, synthetic suppressor T cells protect human beta cell organoids from cytotoxic T cell killing, allowing them to maintain endocrine function (i.e., insulin secretion) in vivo.

## Discussion

These results demonstrate that it is possible to design synthetic suppressor T cells that produce locally targeted immune suppression, such as the ability to block local CAR T cell attack. To generate these synthetic suppressor cells, we engineered conventional CD4^+^ T cells with synNotch induction circuits to produce a diverse range of individual and combinatorial payloads for local suppression of T cell attack. By designing and testing a range of different alternative suppressive circuits, we could explore various effective suppressive solutions and compare these with natural suppressive circuits, such as those in T_reg_ cells (21). T_reg_ cells produce IL-10, TGFβ1, and CD25. The best synthetic suppressor cells shared the common feature of producing a suppressive factor (IL-10, TGFβ, or PD-L1) combined with an inflammatory cytokine sink (CD25, which consumes IL-2). We identified alternative suppressive solutions using synthetic payload combinations. For example, suppressor cell circuits that induced expression of PD-L1, commonly associated with myeloid suppressor cells (e.g., M2 macrophages, tolerogenic dendritic cells) (4), combined with CD25, commonly associated with T_reg_ cells (21), also drove effective suppression.

The manner in which these suppressive signals were produced proved critical to drive strong immune suppression of T cell killing. Optimal circuits had both the production of a suppressive factor, such as TGFβ1, and the cytokine sink, CD25, from the same cell for effective suppression (a feature also observed in T_reg_ cells). CD25 both contributes to increased local IL-2 consumption and drives more proliferation of suppressor cells, which creates a positive feedback loop to subsequently produce more TGFβ1 locally. Expression of large amounts of CD25 could allow suppressor T cells to be more responsive to local IL-2 gradients. By acting both as IL-2 sinks and sources for inhibitory cytokines, suppressor cells could limit local IL-2 gradients and TCR signaling, thereby restricting the ability of T cells to mount a strong immune response (38-41).

Synthetic suppressor T cells have several possible advantages as an alternative therapeutic platform to redirected T_reg_ cell therapies. First, they are derived from human CD4^+^ T cells, a cell type already highly amenable to ex vivo expansion and engineering and in clinical use (42, 43). T_reg_ cell therapies still face major challenges with cell fate instability, limited ex vivo expansion capacity, and programmability of targeting (44-46). By using synNotch circuits to induce suppressive responses in a conventional CD4^+^ T cell, synthetic suppressor T cells are potentially more stable, as they act completely independently of stably maintaining a T_reg_ cell fate. Second, these circuits are modular and therefore highly customizable. Although the native T_reg_ cell circuit could be effective in some situations, the suppressive payloads of synthetic suppressor cells could be customized for specific or more-flexible uses.

This work extends possible ways to engineer suppressor immune cells, which include redirecting native T_reg_ cells (47), using expression of master regulators such as FoxP3 to generate T_reg_ cells (48), or engineering of bespoke suppression programs, as demonstrated here. Each approach will likely be optimal for different classes of suppressive applications. We show that specific payloads can tune the degree to which suppression is targeted to CD4^+^ or CD8^+^ cells, a feature that could be useful for addressing autoimmune diseases driven by different mechanisms. Synthetic suppressor cells might also be tunable in their ability to effectively inhibit other cytotoxic cell types, such as natural killer cells. Moreover, in synthetic suppressor cells, it may be possible to combine immune suppressive payloads with trophic or regenerative payloads that help to simultaneously repair damage induced by autoimmune attack. SynNotch receptors can also be programmed to induce diverse payloads relevant for specific applications or disease indications, such as non-native or orthogonal cytokines, antibodies, or regenerative payloads (49-51).

**Fig. 6.**
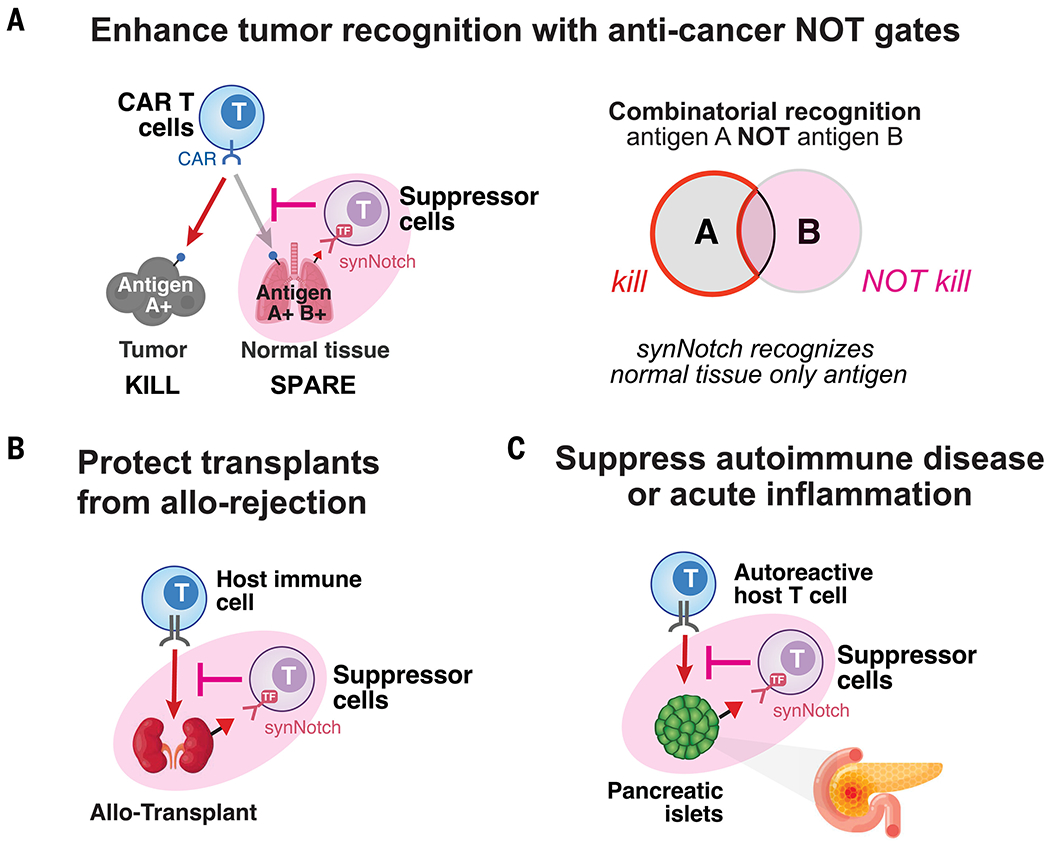
Potential application of synthetic suppressor cells for local immune protection. (**A**) Synthetic suppressor T cells could act as NOT gates to block off-target CAR T cell toxicity in cross-reactive tissues without blocking on-target tumor killing. Suppressor T cells could be directed to off-target tissue (nontumor) using a healthy tissue–specific synNotch to block cytotoxic T cell activity. (**B**) Synthetic suppressor T cells could recognize allogeneic transplants and locally suppress rejection by host immune cells. Local recognition of transplants by suppressor T cells could remodel the transplant microenvironment to improve transplant survival without systemic immunosuppression. (**C**) Synthetic suppressor T cells could locally block autoimmune destruction of tissues (e.g., type 1 diabetes, multiple sclerosis). Suppressor T cells that are directed to protect a target tissue using a tissue-specific synNotch could act locally to prevent or treat autoimmunity.

Synthetic suppressor T cells could be tailored to sculpt immune environments in diverse therapeutic applications, including cancer NOT gates (blocking specific off-target cross-reactions), transplant rejection, and autoimmune disease (Fig. 6). Synthetic suppressor T cells can be directed to specific organs, such as the brain, using synNotch receptors that recognize a tissue-specific antigen to drive local immune suppression (52). In all cases, suppressor T cells could act locally without systemic immune suppression and its associated toxicities. Future studies will need to determine the optimal balance between local suppression and immune privilege in the targeted tissues or transplants. Additional regulatory mechanisms may be needed to tune suppressor T cell survival or payload production to tailor cells for specific therapeutic contexts.

In summary, we reconstituted paracrine cellular immunoregulation using synNotch circuits to generate designer suppressor cells, a potential therapeutic platform for targeted immune suppression. Such synthetic suppressor cells may be used to target immune suppression in a variety of contexts. Synthetic reconstitution of complex immune responses offers a powerful approach to dissect minimal requirements for immune suppression and to design effective therapeutic cell programs.

## Materials and methods

### Viral DNA constructs

Primary human T cells were engineered by lentiviral transduction with constructs cloned into a second-generation 5′ self-inactivating lentiviral backbone (pHR). All lentiviral constructs and sequences are detailed in tables S1 and S2. Suppressor T cells were transduced with either single lentiviral constructs that contain a synNotch receptor, CAR, response element with suppressive payload, or single lentiviral construct that contained both the synNotch receptor, the response element, and suppressive payload. synNotch or CAR was expressed constitutively using mouse PGK promoters. Response elements (induced by synNotch) were controlled by a 5xGAL4 repeat with a minimal cytomegalovirus (CMV) promoter. Suppressive payloads were expressed downstream of the response element alone or with a coexpressed mCherry reporter (“IRES mCherry”). Suppressor T cells inducing combinatorial payloads was generated by cotransducing two lentiviral constructs (one containing a synNotch receptor, response element, and first suppressive payload; the other containing response element and second suppressive payload). For constructs containing only a response element and suppressive payload, a constitutive fluorescent label (“PGK tagBFP”) was cloned for sorting positively transduced T cells. synNotch receptors and CAR T cells were labeled with a Myc or V5 protein tag for sorting positively transduced T cells.

### Primary human T cell isolation and culturing

Human leukapheresis packs were obtained from anonymous donors with approval by the University Institutional Review Board. Primary human CD4^+^ and CD8^+^ T cells were isolated from leukapheresis packs using EasySep kits (Stem Cell Technologies) and frozen in RPMI with 20% human AB serum and 10% dimethyl sulfoxide. Human T cells were thawed and cultured in human T cell media [X-VIVO media (Lonza), 5% human AB serum, 10 mM *N*-acetyl cysteine, 55 μM β mercaptoethanol, 30 U/ml IL-2]. T cells were activated 1 day after thawing with 25 μl anti-CD3/CD28 coated beads [Dynabeads Human T-Activator CD3/CD28 (Gibco)] per 1 × 10^6^ T cells. T cells were infected with lentivirus the day after (2 days after thawing), and the virus was removed from the T cells the following day (3 days after thawing) by centrifugation of T cells at 400*g* for 4 min and removal of lentivirus-containing supernatant and resuspending in human T cell media. T cells were sorted 5 days after thawing for expression of synNotch or CAR by positive staining of a Myc-tag (anti–Myc-tag antibody, 9B11, Alexa Fluor 647 conjugate, Cell Signaling Technology, Cat# 2233) or fluorescent protein expression. T cells were expanded at 1 × 10^6^ cells/ml every day until 10 days after sorting, prior to starting in vitro or in vivo assays.

### Primary human regulatory T cell isolation, CAR transduction, and culturing

Human polyclonal T_reg_ cells were isolated by sorting CD4^+^ (BioLegend, SK3 clone), high CD25^+^ (Thermo Fisher, 4E3 clone), and CD127^−^ (BD Biosciences, HIL-7R-M21 clone) immediately after isolation of primary human CD4^+^ T cells from leukapheresis packs. The same day of sorting, T_reg_ cells were activated with 50 μl anti-CD3/CD28 coated beads [Dynabeads Human T-Activator CD3/CD28 (Gibco)] per 1 × 10^6^ T cells for 7 days and expanded at 1 × 10^6^ cells/ml every day until assay time point using human T cell media with 300 U/ml IL-2. Fixing and intracellular staining (BioLegend Cat# 421403) of isolated T_reg_ cells for FoxP3 (Thermo Fisher, 236A/E7 clone) and Helios (Thermo Fisher, 22F6 clone) was used to test purity of T_reg_ cells before assays. CAR engineered T_reg_ cells were generated by lentiviral transduction of isolated polyclonal T_reg_ cells with anti-CD19 CAR receptor (with CD28 costimulatory domain) at day 7 of expansion. CAR sequence is detailed in table S2. For suppression assays, polyclonal T_reg_ cells were at a density of cultured at 1 million cells/ml with anti-human CD28 antibody (Thermo Fisher, CD28.2 clone) and plate-bound anti-human CD3 antibody (Thermo Fisher, OKT3 clone) for 24 hours before moving cells to a new plate for coculture with target cells and CD8^+^ CART cells.

### Lentivirus production

Lentivirus was produced using Lx293t lentiviral packaging cells (Takara Bio, Cat# 632180) that were seeded in six-well plates at 7 × 10^5^ cells per well and 24 hours later transfected with pHR constructs and pCMV and pMD2.g packaging plasmids using FuGene HD (Promega) following manufacturer’s protocol. Forty-eight hours after transfection, viral supernatant was collected, filtered, and concentrated with LentiX concentrator (Takara Bio, Cat# 631231) for 24 hours before resuspending in human T cell media and use with human T cell cultures.

### Tumor cell culture

Human K562 cells were purchased from ATCC (CCL-243) and cultured in Iscove’s modified Dulbecco’s modified Eagle’s medium with 10% fetal bovine serum and split to 3 × 10^5^ cells/ml every 2 days. Human K562s were engineered to express antigens by lentiviral transduction. Lentivirus was added to the K562 media, removed after 24 hours, and cells were sorted by positive staining 48 hours after removing virus.

### In vitro T cell assays

T cells were labeled with 1:5000 CellTrace CFSE proliferation stain (Molecular Probes) or 1:5000 CellTrace FarRed proliferation stain (Molecular Probes). T cells and target cells were diluted in their respective media to the appropriate density without IL-2 and combined at a 1:1 ratio with equal media of each type. For activation by synNotch activation beads, T cells were mixed with anti–Myc-tag antibody-coated beads (Pierce) were washed three times with hTCM using a magnet before using (10 μl beads per 1 ml media). For assays longer than 3 days, 100 μl of cells and media were diluted in 100 μl of fresh media for a total volume of 200 μl every 3 days. For measurement of secreted cytokines, supernatant was measured by enzyme-linked immuno-sorbent assay (ELISA; R&D Systems). For measurement of intracellular cytokine production, T cells were mixed with target cells and then exposed to GolgiStop (BD biosciences) for 12 hours then fixed before intracellular staining. For measuring intracellular markers during suppression assays, T cells were mixed with target cells as described. After 24 hours, cells were fixed, permeabilized, and stained. All flow cytometry analysis was performed on a BD Fortessa X-20 and analyzed using FlowJo (FlowJo, LLC). For assays with mixed coculture of two different K562 populations, Her2^+^ CD19^+^ K562s were cotransduced with BFP and Her2^+^ CD19^−^ K562s were labeled with BFP and GFP to differentiate populations during flow cytometry analysis. All cell counts were measured by flow cytometry analysis of a fixed volume of the in vitro culture. Cell counts were measured at time of assay set up (day 0), and subsequent measurements were normalized to the initial counts.

### Mouse two-tumor model experiments

All mouse experiments were conducted according to Institutional Animal Care and Use Committee (IACUC)–approved protocols. For tumor experiments, female age 6- to 12-week-old NSG (NOD-*scid* IL2Rgamma^null^) mice were used. K562 tumors were injected in 100 μL phosphate-buffered saline (PBS) subcutaneously into each flank. Tumors were measured by calipers. In all cases, human T cells were injected intravenously by tail vein injection in 100 μL PBS 7 days after injection of tumors.

### Analysis of isolated tumor samples: Flow cytometry

Tumor samples were collected from mice (7 days after T cell injection) and immediately processed. Tumors were minced and digested with of 1 mg/ml collagenase IV, 20 U/ml DNAse IV, and 0.1 mg/ml hyaluronidase V in RPMI for 30 min at 37°C with shaking. The digested cells were washed twice through 70-mm cell strainers then stained for cell surface markers.

### Stem cell–derived beta cells enriched beta cell (eBC) organoid differentiation

Mell INS^GFP/wt^ human embryonic stem cells, obtained from S. J. Micallef and E. G. Stanley (Monash Immunology and Stem Cell Laboratories, Australia) were cultured on mouse embryonic fibroblast (MEFs) in hESC media and passaged using enzymatic digestion. At the beginning of the differentiation, confluent hESC were digested into single-cell suspension using TrypLE and seeded at 5.5 × 10^6^ cells per well in six-well suspension plates in 5.5 ml hPSC media supplemented with 10 ng/ml Activin A (R&D Systems) and 10 ng/ml HeregulinB (Peprotech). The plates were incubated at 37°C and 5% CO_2_ on an orbital shaker at 100 rpm to induce three-dimensional (3D) sphere formation. After 24 hours, the spheres were collected in a 50-ml falcon then washed with RPMI media (Gipco) and resuspended in day 1 media in new six-well suspension plates. Thereafter, media was changed every day at the same time until day 19, as previously described (31), with the exception that all media were enriched with 5 μg/ml Aphidicolin (Cayman Chemical) starting at day 12. On day 19, the spheres were collected and dissociated in a single-cell suspension using Accumax (Sigma-Aldrich) then filtered with a 40-μm cell Strainer (falcon) to ensure the removal of debris or nondigested spheres. The cells were seeded at 4 × 10^6^ cells per well in new six-well suspension plates in the presence or absence of the lentivirus containing CD19 antigen and then placed in orbital shaker at 100 rpm to induce 3D sphere aggregation. The media was changed the next day, then every other day until days 27 to 29.

### eBC organoid

#### In vitro microscopy assays

In vitro assays for suppression of T cell killing of enriched beta cell clusters was performed on an Incucyte Live-Cell Analysis System (Sartorius) or Opera Phenix Plus High-Content Screening System. Enriched beta cell survival was quantified as the integrated GFP signal normalized to the 0 hour time point using the Incucyte Spheroid Analysis Software Module (Sartorius). Caspase 3/7 reporter dye (Incucyte, Cat# 4704) was added at the 0 hour time point at 0.2 μM.

#### In vivo transplantation experiments

NOD-*scid* IL2Rgamma^null^ (NSG) mice were obtained from Jackson Laboratories and bred in our facility. Male and female mice in the age group of 12 to 16 weeks were used in this study and were maintained according to protocols approved by the University of California, San Francisco, IACUC. This study follows all relevant ethical regulations regarding animal research. Mice were anesthetized with isoflurane and transplanted with ~4000 eBCs (~4 × 10^6^ cells) under the kidney capsule. Two weeks after the surgery, the mice were injected intravenously either with (~1 × 10^6^ cells) CD4/CD8 HLA-A2 CAR T cells alone or in combination with (~2 × 10^6^ cells) anti-CD19 synNotch suppressor cells. To assess xenograft luciferase expression, mice were injected intraperitoneally with 15 mg/ml D-luciferin solution (Goldbio Biotechnology, injection volume 200 μl) and then imaged 15 min later using the Xenogen IVIS 200 imaging system (Perkin Elmer). Same-size regions of interest were manually plotted for analysis of all data points to ensure signal consistency within the same experiment.

#### In vivo transplant glucose challenge

For the in vivo glucose challenge experiments, 5 weeks after the surgeries (21 days after T cell injection), male transplanted mice were fasted overnight, and the serum was collected by submandibular bleeding at t0 (before) and t30 (30 min) after intraperitoneal d-glucose injection (1.8 g kg^−1^). Circulating human C-peptide was measure using STELLUX Chemi Human C- peptide ELISA kit (Alpco).

#### In vivo measurement of cytokines

TGFβ1 concentrations were measured by ELISA in relevant tissue types. At 20 days after engraftment of CAR T and synthetic suppressor cells, blood was obtained by submandibular bleeding, spleens were dissected, and eBC grafts were dissected from the kidney. Serum was obtained by permitting coagulation at room temperature for 10 min then centrifuging 10 min at 2000*g*. Protein was extracted from spleens and grafts by mechanical disruption using a needle and syringe in tissue homogenization buffer. Total protein concentration per sample was measured by BCA assay (Thermo Fisher) and TGFβ1 levels were measured using the TGFβ1 Quantikine ELISA kit (R&D Systems) according to the manufacturers’ protocols. Protein concentrations were calculated on the basis of protein standards included in each kit.

### Analysis of isolated transplants

#### Immunohistochemistry

Kidneys containing CD19^+^ eBC organoid transplants were collected for immunohistochemistry and fixed immediately in 10% formalin for 24 hours before preservation at 70% ethanol. Tissues were embedded in paraffin, sectioned, and mounted for staining with anti-human CD19 (ABclonal, ARC0418) antibody at the UCSF Parnassus CoLab.

#### Flow cytometry

Kidneys containing CD19^+^ eBC organoid transplants and spleens from the same mice were collected and immediately processed. Tissue samples were minced and digested with of 1 mg/ml collagenase IV, 20 U/ml DNAse IV, and 0.1 mg/ml hyaluronidase V in RPMI for 30 min at 37°C with shaking. The digested cells were washed twice through 70-μm cell strainers then stained for cell surface markers.

#### Multiplexed ion beam imaging (MIBIscope) sample preparation

Whole kidneys from mice with eBC transplants were isolated and immediately fixed for 24 hours in 4% paraformaldehyde-PBS, washed three times in PBS, and stored in 70% ethanol at −20°C until paraffin processing. Tissue was infiltrated with paraffin wax (Leica/ASP300S) and then embedded into paraffin blocks. Paraffin-embedded tissue blocks (FFPE) were cut at a thickness of 5 mm and after 50 μm cutting was stopped, and cut sections were placed onto a Superfrost plus glass slide (Fisher) and, using standard immunohistochemical methods, stained with anti-human CD19 (ABclonal, Cat#A19013), followed by horse radish peroxidase conjugated anti-rabbit (Cell Signaling Technology) detected with DAB (3,3′-diaminobenzidine, Cell Signaling Technology). The corresponding blocks for tissue sections positive for human cell engraftment were store under vacuum at 4°C, with all blocks processed in this manner.

Serial section of tissue positive for human cell engraftment were mounted on a glass slide and stained for CD19 and mounted onto gold-sputtered microscope slides for multiplexed ion beam imaging processing (Ionpath). Tissue Gold Slides were baked at 70°C overnight and dewaxing and staining were according to Ionpath protocol. Briefly, baked tissue was deparaffinized, dehydrated, and then antigen retrieved using high pH (Dako Target Retrieval) for 40 min at 97°C followed by cooling to 65°C in a Lab Vision PT module. (Thermo Fisher Scientific). Slides were cooled to room temperature for 30 min and washed in two time in TBS-T (Ionpath). Tissues were blocked with 5% donkey serum (DS, Sigma-Aldrich)–TBS-T for 1 hour at room temperature.

Antibody cocktail was resuspended in 5% DS and made to adjusted to a concentration of 0.005 mM EDTA passed through a 0.1 μm centrifugal filter (Millipore). Tissues were stained with antibody cocktail overnight in a humidity chamber at 4°C. The next day, slides were washed twice with TBS-T, followed by PBS, and then antibodies were fixed to tissue by with incubating with 2% glutaldehyde (Electron Microscope Sciences)–PBS for 5 min and neutralized with three volumes of 100 mM Tris pH 8.0. Slides were washed with double-distilled water (2×), 70% ethanol (1×), 80% ethanol (1×), 95% ethanol (2×), and 100% ethanol (2×), air dried for 10 min, and stored under vacuum until MIBI scanning.

#### MIBIscope data acquisition and postprocessing

Imaging was performed using a MIBI-TOF instrument (Ionpath) with a Hyperion ion source. Xe^+^ primary ions were used to sequentially sputter pixels for a given field of view. The following imaging parameters were used: acquisition setting, 80 kHz; field size, 800 mm by 800 mm, 2048 pixels by 2048 pixels; dwell time, 0.25 ms; median gun current on tissue, 10.5 nA Xe^+^.

After image acquisition, single-channel tiffs were extracted from raw bin files through the Angelo Lab’s toffy pipeline (https://github.com/angelolab/toffy/tree/main). Using this pipeline for all subsequent processing steps, single-channel tiffs were mass compensated and normalized to reduce signal interference and retain comparable signal across collected fields of view. Cleaned images were visualized in ImageJ.

### Statistical analysis

All statistical analyses were performed with Prism software version 9.0 (GraphPad), as described in the figures and legends.

## Supplementary Material

Supplementary Materials

MDAR Reproducibility

## Data Availability

All data are available in the manuscript or supplementary materials. Reagents are available from the corresponding author upon reasonable request. Plasmids will be distributed via Addgene.
