# Supplementary material for: Engineering synthetic suppressor T cells that execute locally targeted immunoprotective programs
Source: Science. Author manuscript; Available in PMC 2025 Feb 17. (PMC11831968; doi:10.1126/science.adl4793)
Supplement: Supplementary Materials [file NIHMS2052488-supplement-Supplementary_Materials.pdf]

Science
AAAS

Supplementary Materials for

Engineering synthetic suppressor T cells that execute locally targeted immunoprotective programs

Authors: Nishith R. Reddy[1-2], Hasna Maachi[3,9,10], Yini Xiao[1-3], Milos S. Simic[1-2], Wei Yu[1-2], Yurie Tonai[1-2], Daniela A. Cabanillas[1-2], Ella Serrano-Wu[1-2], Philip T. Pauerstein[1,8], Whitney Tamaki[4], Greg M. Allen[1,5-7], Audrey V. Parent[3], Matthias Hebrok[3,9,10,11], Wendell A. Lim[1-2,6*]

*Corresponding author. Email: Wendell.Lim@ucsf.edu

The PDF file includes:

Materials and Methods
Figs. S1 to S12
Table S1 to S3
References (*32*)

Materials and Methods

Viral DNA Constructs

Primary human T cells were engineered by lentiviral transduction with constructs cloned into a second generation 5' self-inactivating lentiviral backbone (pHR). All lentiviral constructs and sequences are detailed in Tables S1 and S2. Suppressor T cells were transduced with either single lentiviral constructs that contain a synthetic Notch (synNotch) receptor, chimeric antigen receptor (CAR), response element with suppressive payload, or single lentiviral construct that contained both the synNotch receptor, the response element, and suppressive payload. synNotch or CAR was expressed constitutively using mouse PGK promoters. Response elements (induced by synNotch) were controlled by a 5xGAL4 repeat with a minimal CMV promoter. Suppressive payloads were expressed downstream of the response element alone or with a co-expressed mCherry reporter ("IRES mCherry"). Suppressor T cells inducing combinatorial payloads was generated by co-transducing two lentiviral constructs (one containing a synNotch receptor, response element, and first suppressive payload; the other containing response element and second suppressive payload). For constructs containing only a response element and suppressive payload, a constitutive fluorescent label ("PGK tagBFP") was cloned for sorting positively transduced T cells. synNotch receptors and CAR T cells were labeled with a Myc or V5 protein tag for sorting positively transduced T cells.

Primary Human T Cell Isolation and Culturing

Human leukapheresis packs were obtained from anonymous donors with approval by the University Institutional Review Board. Primary human CD4+ and CD8+ T cells were isolated from leukapheresis packs using EasySep kits (Stem Cell Technologies) and frozen in RPMI with 20% human AB serum and 10% DMSO. Human T cells were thawed and cultured in human T cell media (X-VIVO media [Lonza], 5% human AB serum, 10 mM n-acetyl cysteine, 55 μM β mercaptoethanol, 30 U/mL IL-2). T cells are activated one day after thawing with 25 μL anti-CD3/CD28 coated beads (Dynabeads Human T-Activator CD3/CD28 [Gibco]) per 1e6 T cells. T cells were infected with lentivirus the day after (2 days after thawing) and the virus was removed from the T cells the following day (3 days after thawing) by centrifugation of T cells at 400xG for 4 minutes and removal of lentivirus-containing supernatant and resuspending in human T cell media. T cells were sorted 5 days after thawing for expression of synNotch or CAR by positive staining of a Myc-tag (anti-Myc-tag antibody, 9B11, Alexa Fluor 647 conjugate, Cell Signaling Technology, Cat# 2233) or fluorescent protein expression. T cells were expanded at 1e6 cells/mL every day until 10 days post-sort prior to starting in vitro or in vivo assays.

Primary Human Regulatory T Cell Isolation, CAR transduction, and Culturing

Human polyclonal regulatory T cells (Tregs) were isolated by sorting CD4+ (Biolegend, SK3 clone) CD25++ (Thermo Fisher, 4E3 clone) CD127- (BD Biosciences, HIL-7R-M21 clone) immediately after isolation of primary human CD4+ T cells from leukapheresis packs. The same day of sorting, Tregs were activated with 50 μL anti-CD3/CD28 coated beads (Dynabeads

Human T-Activator CD3/CD28 [Gibco]) per 1e6 T cells for 7 days and expanded at 1e6 cells/mL every day until assay timepoint using human T cell media with 300 U/mL IL-2. Fixing and intracellular staining (Biolegend Cat # 421403) of isolated Tregs for FoxP3 (Thermo Fisher, 236A/E7 clone) and Helios (Thermo Fisher, 22F6 clone) was used to test purity of Tregs prior to assays. CAR engineered Tregs were generated by lentiviral transduction of isolated polyclonal Tregs with anti-CD19 CAR receptor (with CD28 co-stimulatory domain) at day 7 of expansion. CAR receptor sequence is detailed in Table 2. For suppression assays, polyclonal Tregs were at a density of cultured at 1 million cells/mL with anti-human CD28 antibody (Thermo Fisher, CD28.2 clone) and plate bound anti-human CD3 antibody (Thermo Fisher, OKT3 clone) for 24 hours prior to moving cells to a new plate for co-culture with target cells and CD8+ CAR T cells.

Lentivirus Production

Lentivirus was produced using Lx293t lentiviral packaging cells (Takara bio, Cat# 632180) that were seeded in 6-well plates at 7e5 cells/well and 24 hours later transfected with pHR constructs and pCMV and pMD2.g packaging plasmids using FuGene HD (Promega) following manufacturer's protocol. 48 hours after transfection viral supernatant was collected, filtered, and concentrated with LentiX concentrator (Takara bio, Cat# 631231) for 24 hours prior to resuspending in human T cell media and use with human T cell cultures.

Tumor Cell Culture

Human K562 cells were purchased from ATCC (CCL-243) and cultured in Iscove Modified Dulbecco's Modified Eagle Medium with 10% FBS and split to 3e5 cells/mL every 2 days. Human K562s were engineered to express antigens by lentiviral transduction. Lentivirus was added to the K562 media, removed after 24 hours, and cells were sorted by positive staining 48 hours after removing virus.

In-Vitro T Cell Assays

T cells were labeled with 1:5000 CellTrace CFSE proliferation stain (Molecular Probes) or 1:5000 CellTrace FarRed proliferation stain (Molecular Probes). T cells and target cells were diluted in their respective media to the appropriate density without IL-2 and combined at a 1:1 ratio with equal media of each type. For activation by synNotch activation beads, T cells were mixed with anti-Myc-tag antibody-coated beads (Pierce) were washed 3 times with hTCM using a magnet before using (10 uL beads/1 mL media). For assays longer than 3 days, 100 uL of cells and media were diluted in 100 uL of fresh media for a total volume of 200 uL every 3 days. For measurement of secreted cytokines, supernatant was measured by ELISA (R&D systems). For measurement of intracellular cytokine production, T cells were mixed with target cells and then exposed to GolgiStop (BD biosciences) for 12 hours then fixed prior to intracellular staining. For measuring intracellular markers during suppression assays, T cells were mixed with target cells as described. After 24 hours, cells were fixed, permeabilized and stained. All flow cytometry analysis was performed on a BD Fortessa X-20 and analyzed using FlowJo (FlowJo, LLC). For

assays with mixed co-culture of two different K562 populations, Her2+ CD19+ K562s were co-transduced with BFP and Her2+ CD19- K562s were labeled with BFP and GFP to differentiate populations during flow cytometry analysis. All cell counts were measured by flow cytometry analysis of a fixed volume of the in vitro culture. Cell counts were measured at time of assay set up (day 0) and subsequent measurements were normalized to the initial counts.

Mouse Two Tumor Model Experiments

All mouse experiments were conducted according to Institutional Animal Care and Use Committee (IACUC)–approved protocols. For tumor experiments, female age 6 to 12 week old NSG (NOD-*scid* IL2Rgamma^{null}) mice were used. K562 tumors were injected in 100 uL PBS subcutaneously into each flank. Tumors were measured by calipers. In all cases, human T cells were injected intravenously by tail vein injection in 100 μL PBS 7 days after injection of tumors.

Analysis of Isolated Tumor Samples: Flow Cytometry

Tumor samples were collected from mice (7 days after T cell injection) and immediately processed. Tumors were minced and digested with of 1 mg/mL collagenase IV, 20 U/mL DNAse IV, and 0.1 mg/mL hyaluronidase V in RPMI for 30 min at 37°C with shaking. The digested cells were washed twice through 70 μm cell strainers then stained for cell surface markers.

Stem Cell-Derived Beta Cells Enriched Beta Cell (eBC) Organoid Differentiation

Mell INS$^{GFP/wt}$ human embryonic stem cells, obtained from S.J. Micallef and E.G. Stanley (Monash Immunology and Stem Cell Laboratories, Australia) were cultured on embryonic fibroblast (MEFs) in hESC media and passaged using enzymatic digestion. At the beginning of the differentiation, confluent hESC were digested into single cell suspension using TrypLE and seeded at 5.5 x 10^6 cell/well in a 6 well suspension plates in 5.5 ml hPSC media supplemented with 10 ng/ml Activin A (R&D Systems) and 10 ng/ml HeregulinB (Peprotech). The plates were incubated at 37°C and 5% CO_2 on an orbital shaker at 100 rpm to induce 3D sphere formation. After 24 hours, the spheres were collected in a 50 ml falcon then washed with RPMI media (Gipco) and resuspended in day 1 media in a new 6 well suspension plates. Thereafter media was changed every day at the same time until day 19 as previously described (*32*) with the exception that all media were enriched with 5 μg/ml Aphidicolin (Cayman Chemical) starting at day 12. On day 19, the spheres were collected and dissociated in a single cell suspension using Accumax (Sigma-Aldrich) then filtered with a 40-μm cell Strainer (falcon) to ensure the removal of debris or non-digested spheres. The cells were seeded at 4 x 10^6 cell/well in a new 6 well suspension plates in the presence or absence of the lentivirus containing CD19 antigen and then placed in orbital shaker at 100 rpm to induce 3D sphere aggregation. The media was changed the following day, then every other day until d27-29.

eBC Organoid: In-Vitro Microscopy Assays

In vitro assays for suppression of T cell killing of enriched beta cell clusters was performed on an Incucyte Live-Cell Analysis System (Sartorius) or Opera Phenix Plus High-Content Screening System. Enriched beta cell survival was quantified as the integrated GFP signal normalized to the 0 hour timepoint using the Incucyte Spheroid Analysis Software Module (Sartorius). Caspase 3/7 reporter dye (Incucyte, Cat# 4704) was added at the 0 hour timepoint at 0.2 μM.

eBC Organoid: In-Vivo Transplantation Experiments

NOD-*scid* IL2Rgamma^{null} (NSG) mice were obtained from Jackson Laboratories and bred in our facility. Male and female mice between the age group of 12–16 weeks were used in this study and were maintained according to protocols approved by the University of California, San Francisco, Institutional Animal Care and Use Committee. This study follows all relevant ethical regulations regarding animal research. Mice were anesthetized with isoflurane and transplanted with ~4000 eBCs (~4×10^6 cells) under the kidney capsule. Two weeks after the surgery, the mice were injected intravenously either with (~1×10^6 cells) CD4/CD8 HLA-A2 CAR T cells alone or in combination with (~2×10^6 cells) anti-CD19 synNotch suppressor cells. To assess xenograft luciferase expression, mice were injected intraperitoneally with 15 mg/ml D-luciferin solution (Goldbio Biotechnology, injection volume 200 μl) and then imaged 15 minutes later using the Xenogen IVIS 200 imaging system (Perkin Elmer). Same size regions of interest were manually plotted for analysis of all data points to ensure signal consistency within the same experiment.

eBC Organoid: In-Vivo Transplant Glucose Challenge

For the in vivo glucose challenge experiments, five weeks after the surgeries (21 days after T cell injection), male transplanted mice were fasted overnight, and the serum was collected by sub-mandibular bleeding at t0 (before) and t30 (30 min) following intraperitoneal D-Glucose injection (1.8 g kg^{-1}). Circulating human C-peptide was measure using STELLUX® Chemi Human C-peptide ELISA kit (Alpco).

eBC Organoid: In-Vivo Measurement of Cytokines

TGFB1 concentrations were measured by ELISA in relevant tissue types. At 20 days after engraftment of CAR-T and synthetic suppressor cells, blood was obtained by submandibular bleeding, spleens were dissected, and eBC grafts were dissected from the kidney. Serum was obtained by permitting coagulation at room temperature for 10 min, then centrifuging 10 min at 2000xg. Protein was extracted from spleens and grafts by mechanical disruption using a needle and syringe in tissue homogenization buffer. Total protein concentration per sample was measured by BCA assay (Thermo Fisher) and TGFB1 levels were measured using the TGFB1 Quantikine ELISA kit (R&D Systems) according to the manufacturers' protocols. Protein concentrations were calculated based on protein standards included in each kit.

Analysis of Isolated Transplants: Immunohistochemistry

Kidney containing CD19+ eBC organoid transplants were collected for IHC and fixed immediately in 10% formalin for 24 hours prior to preservation at 70% EtOH. Tissues were embedded in paraffin, section, and mounted for staining with anti-human CD19 (Abclonal, ARC0418) antibody at the UCSF Parnassus CoLab.

Analysis of Isolated Transplants: Flow Cytometry

Kidneys containing CD19+ eBC organoid transplants and spleens from the same mice were collected and immediately processed. Tissue samples were minced and digested with of 1 mg/mL collagenase IV, 20 U/mL DNAse IV, and 0.1 mg/mL hyaluronidase V in RPMI for 30 min at 37°C with shaking. The digested cells were washed twice through 70 μm cell strainers then stained for cell surface markers.

Analysis of Isolated Transplants: Multiplex Ion Beam Imaging (MIBIscope) Sample Preparation

Transplanted whole kidneys were isolated and immediately fixed for 24 hrs in 4% paraformaldehyde-PBS, washed 3 times in PBS and stored to 70% Ethanol at -20°C until paraffin processing. Tissue was infiltrated with paraffin wax (Leica /ASP300S) and then embedded into paraffin blocks. Paraffin embedded tissue blocks (FFPE) were cut at a thickness of 5mm and after 50 μm cutting was stopped and cut sections were placed onto a Superfrost plus glass slide (Fisher) and using standard immuno-histochemical methods stained with anti-human CD19 (Abclonal, Cat#A19013), followed by horse radish peroxidase conjugated anti-rabbit (Cell Signaling Technology) detected with DAB (3,3'-diaminobenzidine, Cell Signaling Technology). The corresponding blocks for tissue sections positive for human cell engraftment were store under vacuum at 4°C with all blocks processed in this manner.

Serial section of tissue positive for human cell engraftment were mounted on a glass slide and stained for CD19 and mounted onto gold-sputtered microscope slides for Multiplex Ion Beam Imaging processing (IonPath). Tissue Gold Slides were baked at 70˚C overnight and dewaxing and staining were according to Ionpath protocol. Briefly, baked tissue was deparaffinized, dehydrated, and then antigen retrieved using high pH (Dako Target Retrieval) for 40 min at 97˚C followed by cooling to 65˚C in a Lab Vision PT module. (Thermo Fisher Scientific). Slides were cooled to room temperature for 30 min and washed in 2 time in TBS-T (Ionpath). Tissues were blocked with 5% donkey serum (DS, Sigma-Aldrich)-TBS-T for 1 hour at room temperature. Antibody cocktail was resuspended in 5% DS and made to adjusted to a concentration of 0.005 mM EDTA passed through a 0.1 μm centrifugal filter (Millipore). Tissue were stained with antibody cocktail overnight in a humidity chamber at 4˚C. The following day, slides were washed twice with TBS-T, followed by PBS and then antibodies were fixed to tissue by with incubating with 2% Glutaldehyde (Electron Microscope Sciences)-PBS for 5 min and neutralized with 3 volumes of 100 mM Tris pH 8.0. Slides were washed with ddH2O (2x), 70% ethanol (1x),

80% ethanol (1x), 95% ethanol (2x), and 100% ethanol (2x), air dried for 10 min and stored under vacuum until MIBI scanning.

Analysis of Isolated Transplants: MIBIscope Data Acquisition and Post-Processing
Imaging was performed using a MIBI-TOF instrument (IonPath) with a Hyperion ion source. Xe+ primary ions were used to sequentially sputter pixels for a given field of view. The following imaging parameters were used: acquisition setting: 80 kHz; field size: 800 x 800 mm, 2048 x 2048 pixels; dwell time: 0.25 ms; median gun current on tissue: 10.5 nA Xe+.

After image acquisition, single channel tiffs were extracted from raw bin files via the Angelo Lab's toffy pipeline (https://github.com/angelolab/toffy/tree/main). Using this pipeline for all subsequent processing steps, single channel tiffs were mass compensated and normalized to reduce signal interference and retain comparable signal across collected FOVs. Cleaned images were visualized in ImageJ.

Statistical Analysis
All statistical analyses were performed with Prism software version 9.0 (GraphPad) as described in the figures and legends.

Table S1.

**Name (all in pHR backbone)**	**Order of Elements (:: indicates same transcript)**
anti-CD19 synNotch	pGK promoter, Kozak sequence, CD8 signal sequence::Myc-tag::anti-CD19-scFv::synNotch GAL4VP64
anti-Her2 CAR	pGK promoter, Kozak sequence, CD8 signal sequence::Myc-tag::anti-Her2-scFv::CD8a TMD:: 41BB domain::CD3zeta
anti-HLA-A2 CAR	pGK promoter, Kozak sequence, CD8 signal sequence::Myc-tag::anti-HLA-A2-scFv::CD8a TMD:: CD28 domain::CD3zeta
anti-CD19 iCAR	pGK promoter, Kozak sequence, CD8 signal sequence::V5-tag::anti-CD19-scFv:: CD8a TMD:: PD-1 domain
GAL4UAS IRES mCherry (No Payload Control)	GAL4UAS, IRES, mCherry
GAL4UAS hIL-10 IRES mCherry	GAL4UAS, human IL-10, IRES, mCherry

GAL4UAS TGFβ1 IRES mCherry	GAL4UAS, active TGFb1, IRES, mCherry
GAL4UAS hIL-35 IRES mCherry	GAL4UAS, human IL-35, IRES, mCherry
GAL4UAS hCD25 IRES mCherry	GAL4UAS, human CD25, IRES, mCherry
GAL4UAS sTNFαR IRES mCherry	GAL4UAS, human sTNFαR, IRES, mCherry
GAL4UAS hPD-L1 IRES mCherry	GAL4UAS, human PD-L1, IRES, mCherry
GAL4UAS hCTLA4 IRES mCherry	GAL4UAS, human CTLA4, IRES, mCherry
GAL4UAS hCD39 IRES mCherry	GAL4UAS, human hCD39, IRES, mCherry
GAL4UAS hIL-2 IRES mCherry	GAL4UAS, human IL-2, IRES, mCherry
SFFV CD19-ligand	SFFV promoter, Kozak sequence, IgKappa signal sequence::CD19 ECD::PDGFRb TMD
SFFV Her2-BFP-ligand	SFFV promoter, Kozak sequence, IgKappa signal sequence::Her2 ECD TMD::BFP

Constituent components of pHR lentiviral vectors

Table S2.

Sequences of components referenced in Supplementary Table 1.

**Element Name**	**Sequence**
Anti-CD19-scFv	DIQMTQTTSSLSASLGDRVTISCRASQDISKYLNWYQQKPDGTV<br>KLLIYHTSRLHSGVPSRFSGSGSGTDYSLTISNLEQEDIATYFCQQ<br>GNTLPYTFGGGTKLEITGGGGSGGGGSGGGGSEVKLQESGPGLV<br>APSQSLSVTCTVSGVSLPDYGVSWIRQPPRKGLEWLGVIWGSETT<br>YYNSALKSRLTIIKDNSKSQVFLKMNSLQTDDTAIYYCAKHYYYG<br>GSYAMDYWGQGTSVTVSS
CD8 signal sequence	MALPVTALLLPLALLLHAARP
synNotch GAL4VP64	ILDYSFTGGAGRDIPPPQIEEACELPECQVDAGNKVCNLQCNNHA<br>CGWDGGDCSLNFNDPWKNCTQSLQCWKYFSDGHCDSQCNSAG<br>CLFDGFDCQLTEGQCNPLYDQYCKDHFSDGHCDQGCNSAECEW<br>DGLDCAEHVPERLAAGTLVLVVLLPPDQLRNNSFHFLRELSHVL<br>HTNVVFKRDAQGQQMIFPYYGHEEELRKHPIKRSTVGWATSSLL<br>PGTSGGRQRRELDPMDIRGSIVYLEIDNRQCVQSSSQCFQSATDV<br>AAFLGALASLGSLNIPYKIEAVKSEPVEPPLPSQLHLMYVAAAAF<br>VLLFFVGCGVLLSRKRRRMKLLSSIEQACDICRLKKLKCSKEKPK<br>CAKCLKNNWECRYSPKTKRSPLTRAHLTEVESRLERLEQLFLLIF<br>PREDLDMILKMDSLQDIKALLTGLFVQDNVNKDAVTDRLASVET<br>DMPLTLRQHRISATSSSEESSNKGQRQLTVSAAAGGSGGSGGSDA<br>LDDFDLDMLGSDALDDFDLDMLGSDALDDFDLDMLGSDALDDF<br>DLDMLGS

GAL4UAS (5x Gal4 response elements with mCMV promoter)	GGAGCACTGTCCTCCGAACGTCGGAGCACTGTCCTCCGAACGT CGGAGCACTGTCCTCCGAACGTCGGAGCACTGTCCTCCGAACG GAGCATGTCCTCCGAACGTCGGAGCACTGTCCTCCGAACGACT AGTTAGGCGTGTACGGTGGGAGGCCTATATAAGCAGAGCTCGT TTAGTGAACCGTCAGATCGCCTGGAGACGCCATCCACGCTGTTT TGACCTCCATAGAAGACACCGGGACCGATCCAGC
SFFV promoter (constitutive)	CCGATAAAATAAAAGATTTTATTTAGTCTCCAGAAAAAGGGG GGAATGAAAGACCCCACCTGTAGGTTTGGCAAGCTAGCTGCA GTAACGCCATTTTGCAAGGCATGGAAAAATACCAAACCAAGA ATAGAGAAGTTCAGATCAAGGGCGGGTACATGAAAATAGCTA ACGTTGGGCCAAACAGGATATCTGCGGTGAGCAGTTTCGGCCC CGGCCCGGGGCCAAGAACAGATGGTCACCGCAGTTTCGGCCCC GGCCCGAGGCCAAGAACAGATGGTCCCCAGATATGGCCCAACC CTCAGCAGTTTCTTAAGACCCATCAGATGTTTCCAGGCTCCCCC AAGGACCTGAAATGACCCTGCGCCTTATTTGAATTAACCAATC AGCCTGCTTCTCGCTTCTGTTCGCGCGCTTCTGCTTCCCGAGCT CTATAAAAGAGCTCACAACCCCTCACTCGGCGCGCCAGTCCTC CGACAGACTGAGTCGCCCGGG
pGK promoter (constitutive)	GGGTAGGGGAGGCGCTTTTCCCAAGGCAGTCTGGAGCATGC GCTTTAGCAGCCCCGCTGGGCACTTGGCGCTACACAAGTGG CCTCTGGCCTCGCACACATTCCACATCCACCGGTAGGCGCC AACCGGCTCCGTTCTTTGGTGGCCCCTTCGCGCCACCTTCTA CTCCTCCCCTAGTCAGGAAGTTCCCCCCCGCCCCGCAGCTCG CGTCGTGCAGGACGTGACAAATGGAAGTAGCACGTCTCACT AGTCTCGTGCAGATGGACAGCACCGCTGAGCAATGGAAGCG GGTAGGCCTTTGGGGCAGCGGCCAATAGCAGCTTTGCTCCTT CGCTTTCTGGGCTCAGAGGCTGGGAAGGGGTGGGTCCGGGG GCGGGCTCAGGGGCGGGCTCAGGGGCGGGGCGGGCGCCCG AAGGTCCTCCGGAGGCCCGGCATTCTGCACGCTTCAAAAGC GCACGTCTGCCGCGCTGTTCTCCTCTTCCTCATCTCCGGGCC TTTCG
Kozak sequence	GCCGCCACC
Myc-tag	EQKLISEEDL
V5-tag	GKPIPNPLLGLDST
IRES	CCCCTCTCCCTCCCCCCCCCCTAACGTTACTGGCCGAAGCCG CTTGGAATAAGGCCGGTGTGCGTTTGTCTATATGTTATTTTC CACCATATTGCCGTCTTTTGGCAATGTGAGGGCCCGGAAAC CTGGCCCTGTCTTCTTGACGAGCATTCCTAGGGGTCTTTCCC CTCTCGCCAAAGGAATGCAAGGTCTGTTGAATGTCGTGAAG GAAGCAGTTCCTCTGGAAGCTTCTTGAAGACAAACAACGTC TGTAGCGACCCTTTGCAGGCAGCGGAACCCCCCACCTGGCG ACAGGTGCCTCTGCGGCCAAAAGCCACGTGTATAAGATACA CCTGCAAAGGCGGCACAACCCCAGTGCCACGTTGTGAGTTG GATAGTTGTGGAAAGAGTCAAATGGCTCTCCTCAAGCGTAT TCAACAAGGGGCTGAAGGATGCCCAGAAGGTACCCCATTGT ATGGGATCTGATCTGGGGCCTCGGTGCACATGCTTTACATGT GTTTAGTCGAGGTTAAAAAACGTCTAGGCCCCCCGAACCA CGGGGACGTGGTTTTCCTTTGAAAAACACGATGATAA

tagBFP	MSELIKENMHMKLYMEGTVDNHHFKCTSEGEGKPYEGTQTMR<br>IKVVEGGPLPFAFDILATSFLYGSKTFINHTQGIPDFFKQSFPEGF<br>TWERVTTYEDGGVLTATQDTSLQDGCLIYNVKIRGVNFTSNGP<br>VMQKKTLGWEAFTETLYPADGGLEGRNDMALKLVGGSHLIAN<br>IKTTYRSKKPAKNLKMPGVYYVDYRLERIKEANNETYVEQHEV<br>AVARYCDLPSKLGHKLN
mCherry	MVSKGEEDNMAIIKEFMRFKVHMEGSVNGHEFEIEGEGEGRPY<br>EGTQTAKLKVTKGGPLPFAWDILSPQFMYGSKAYVKHPADIPD<br>YLKLSFPEGFKWERVMNFEDGGVVTVTQDSSLQDGEFIYKVKL<br>RGTNFPSDGPVMQKKTMGWEASSERMYPEDGALKGEIKQRLK<br>LKDGGHYDAEVKTTYKAKKPVQLPGAYNVNIKLDITSHNEDY<br>TIVEQYERAEGRHSTGGMDELYK
CD19 ECD::<br>PDGFRb TMD<br>(CD19 Ligand)	RPEEPLVVKVEEGDNAVLQCLKGTSDGPTQQLTWSRESPLKP<br>FLKLSLGLPGLGIHMRPLAIWLFIFNVSQQMGGFYLCQPGPPS<br>EKAWQPGWTVNVEGSGELFRWNVSDLGGLGCGLKNRSSEG<br>PSSPSGKLMSPKLYVWAKDRPEIWEGEPPCLPPRDSLNQSLSQ<br>DLTMAPGSTLWLSCGVPPDSVSRGPLSWTHVHPKGPKSLLSL<br>ELKDDRPARDMWVMETGLLLPRATAQDAGKYYCHRGNLTM<br>SFHLEITARPVLWHWLLRTGGWKNAVGQDTQEVIVVPHSLPF<br>KVVVISAILALVVLTIISLIILIMLWQKKPR
Her2 ECD TMD:: BFP<br>(Her2 Ligand)	TQVCTGTDMKLRLPASPETHLDMLRHLYQGCQVVQGNLELT<br>YLPTNASLSFLQDIQEVQGYVLIAHNQVRQVPLQRLRIVRGTQ<br>LFEDNYALAVLDNGDPLNNTTPVTGASPGGLRELQLRSLTEIL<br>KGGVLIQRNPQLCYQDTILWKDIFHKNNQLALTLIDTNRSRAC<br>HPCSPMCKGSRCWGESSEDCQSLTRTVCAGGCARCKGPLPTD<br>CCHEQCAAGCTGPKHSDCLACLHFNHSGICELHCPALVTYNT<br>DTFESMPNPEGRYTFGASCVTACPYNYLSTDVGSCTLVCPLHN<br>QEVTAEDGTQRCEKCSKPCARVCYGLGMEHLREVRAVTSANI<br>QEFAGCKKIFGSLAFLPESFDGDPASNTAPLQPEQLQVFETLEE<br>ITGYLYISAWPDSLPDLSVFQNLQVIRGRILHNGAYSLTLQGLG<br>ISWLGLRSLRELGSGLALIHHNTHLCFVHTVPWDQLFRNPHQA<br>LLHTANRPEDECVGEGLACHQLCARGHCWGPGPTQCVNCSQ<br>FLRGQECVEECRVLQGLPREYVNARHCLPCHPECQPQNGSVT<br>CFGPEADQCVACAHYKDPPFCVARCPSGVKPDLSYMPIWKFP<br>DEEGACQPCPINCTHSCVDLDDKGCPAEQRASPLTSIISAVVGI<br>LLVVVLGVVFGILIGSGSGSGS MSELIKENMHMKLYMEGTVD<br>NHHFKCTSEGEGKPYEGTQTMRIKVVEGGPLPFAFDILATSFLY<br>GSKTFINHTQGIPDFFKQSFPEGFTWERVTTYEDGGVLTATQDT<br>SLQDGCLIYNVKIRGVNFTSNGPVMQKKTLGWEAFTETLYPAD<br>GGLEGRNDMALKLVGGSHLIANIKTTYRSKKPAKNLKMPGVY<br>YVDYRLERIKEANNETYVEQHEVAVARYCDLPSKLGHKLN
anti-Her2-scFv::CD8a<br>TMD:: 41BB<br>domain::CD3zeta<br>(anti-Her2 CAR)	DIQMTQSPSSLSASVGDRVTITCRASQDVNTAVAWYQQKPGKA<br>PKLLIYSASFLESGVPSRFSGSGSGTDFTLTISSLQPEDFATYYCQ<br>QHYTTPPTFGQGVKVEIKRTGSTSGSGKPGSGEGSEVQLVESGG<br>GLVQPGGSLRLSCAASGFNIKDTYIHWVRQAPGKGLEWVARIY<br>PTNGYTRYADSVKGRFTISADTSKNTAYLQMNSLRAEDTAVYY<br>CSRWGGDGFYAMDVWGQGTLVTVSSGSTTTPAPRPPTPAPTIAS<br>QPLSLRPEACRPAAGGAVHTRGLDFACDIYIWAPLAGTCGVLLL<br>SLVITLYCSLKRGRKKLLYIFKQPFMRPVQTTQEEDGCSCRFPEE<br>EEGGCEL RVKFSRSADAPAYKQGQNQLYNELNLGRREEYDVLD<br>KRRGRDPEMGGKPRRKNPQEGLYNELQKDKMAEAYSEIGMKG<br>ERRRGKGHDGLYQGLSTATKDTYDALHMQALPPR

anti-HLA-A2-scFv::CD8a TMD:: CD28 domain::CD3zeta (anti-HLA-A2 CAR)	EISEVQLVESGGGLVQPGGSLRLSCAASGYTFTDYYIHWVRQAP GKGLEWMAWISPHTGGTIYADSVKGRFTISADTSKNTAYLQMN SLRAEDTAVYYCARGPDDWNDGDAFDIWGQGTLVTVSSGGGG SGGGGSGGGGSSDIQMTQSPSSLSASVGDRVTITCKSSQSVLYSS NNENFLAWYQQKPGKAPKLLIYWASTRESGVPSRFSGSRSGTDF TLTISSLQPEDFATYYCQQYYSTPITFGQGTKVEIKESKYGPPCPP CPMFWVLVVVGGVLACYSLLVTVAFIIFWVRSKRSRLLHSDYM NMTPRRPGPTRKHYQPYAPPRDFAAYRSRVKFSRSADAPAYQQ GQNQLYNELNLGRREEYDVLDKRRGRDPEMGGKPRRKNPQEG LYNELQKDKMAEAYSEIGMKGERRRGKGHDGLYQGLSTATKD TYDALHMQALPPR
anti-CD19-scFv:: CD8a TMD:: PD-1 domain (anti-CD19 iCAR)	DIQMTQTTSSLSASLGDRVTISCRASQDISKYLNWYQQKPDGTV KLLIYHTSRLHSGVPSRFSGSGSGTDYSLTISNLEQEDIATYFCQQ GNTLPYTFGGGTKLEITGGGGSGGGGSGGGGSEVKLQESGPGLV APSQSLSVTCTVSGVSLPDYGVSWIRQPPRKGLEWLGVIWGSET TYYNSALKSRLTIIKDNSKSQVFLKMNSLQTDDTAIYYCAKHYY YGGSYAMDYWGQGTSVTVSSTTTPAPRPPTPAPTIASQPLSLRPE ACRPAAGGAVHTRGLDFACDIYIWAPLAGTCGVLLLSLVITLYCI CSRAARGTIGARRTGQPLKEDPSAVPVFSVDYGELDFQWREKT PEPPVPCVPEQTEYATIVFPSGMGTSSPARRGSADGPRSAQPLRP EDGHCSWPL
anti-CD19-scFv:: CD8a TMD:: CD28 domain (anti-CD19 CAR)	DIQMTQTTSSLSASLGDRVTISCRASQDISKYLNWYQQKPDGTVK LLIYHTSRLHSGVPSRFSGSGSGTDYSLTISNLEQEDIATYFCQQGN TLPYTFGGGTKLEITGGGGSGGGGSGGGGSEVKLQESGPGLVAPS QSLSVTCTVSGVSLPDYGVSWIRQPPRKGLEWLGVIWGSETTYYN SALKSRLTIIKDNSKSQVFLKMNSLQTDDTAIYYCAKHYYYGGSY AMDYWGQGTSVTVSSTTTPAPRPPTPAPTIASQPLSLRPEACRPAA GGAVHTRGLDFACDIYIWAPLAGTCGVLLLSLVITLYCRSKRSRLL HSDYMNMTPRRPGPTRKHYQPYAPPRDFAAYRSRVKFSRSADAPA YQQGQNQLYNELNLGRREEYDVLDKRRGRDPEMGGKPRRKNPQE GLYNELQKDKMAEAYSEIGMKGERRRGKGHDGLYQGLSTATKDT YDALHMQALPPR
hIL-10	MHSSALLCCLVLLTGVRASPGQGTQSENSCTHFPGNLPNMLRDL RDAFSRVKTFFQMKDQLDNLLLKESLLEDFKGYLGCQALSEMIQ FYLEEVMPQAENQDPDIKAHVNSLGENLKTLRLRLRRCHRFLPC ENKSKAVEQVKNAFNKLQEKGIYKAMSEFDIFINYIEAYMTMKIRN
Active TGFβ1	MPPSGLRLLPLLLPLPWLLVLTPGRPAAGLSTCKTIDMELVKRKRIE AIRGQILSKLRLASPPSQGEVPPGPLPEAVLALYNSTRDRVAGESAD PEPEPEADYYAKEVTRVLMVDRNNAIYEKTKDISHSIYMFFNTSDIR EAVPEPPLLSRAELRLQRLKSSVEQHVELYQKYSNNSWRYLGNRLL TPTDTPEWLSFDVTGVVRQWLNQGDGIQGFRFSAHSSSDSKDNKLH VEINGISPKRRGDLGTIHDMNRPFLLLMATPLERAQHLHSSRHRRAL DTNYCFSSTEKNCCVRQLYIDFRKDLGWKWIHEPKGYHANFCLGPC PYIWSLDTQYSKVLALYNQHNPGASASPCCVPQALEPLPIVYYVGR KPKVEQLSNMIVRSCKCS
hIL-35	MTPQLLLALVLWASCPPCSGRKGPPAALTLPRVQCRASRYPIAVDCS WTLPPAPNSTSPVSFIATYRLGMAARGHSWPCLQQTPTSTSCTITDVQ LFSMAPYVLNVTAVHPWGSSSSFVPFITEHIIKPDPPEGVRLSPLAERQ LQVQWEPPGSWPFPEIFSLKYWIRYKRQGAARFHRVGPIEATSFILRA VRPRARYYVQVAAQDLTDYGELSDWSLPATATMSLGKGGGSGGGS GGGSGGGSRNLPVATPDPGMFPCLHHSQNLLRAVSNMLQKARQTLE FYPCTSEEIDHEDITKDKTSTVEACLPLELTKNESCLNSRETSFITNGSC LASRKTSFMMALCLSSIYEDLKMYQVEFKTMNAKLLMDPKRQIFLD QNMLAVIDELMQALNFNSETVPQKSSLEEPDFYKTKIKLCILLHAFRIR AVTIDRVMSYLNAS

hCD25	MDSYLLMWGLLTFIMVPGCQAELCDDDPPEIPHATFKAMAYKEGT MLNCECKRGFRRIKSGSLYMLCTGNSSHSSWDNQCQCTSSATRNTT KQVTPQPEEQKERKTTEMQSPMQPVDQASLPGHCREPPPWENEATE RIYHFVVGQMVYYQCVQGYRALHRGPAESVCKMTHGKTRWTQP QLICTGEMETSQFPGEEKPQASPEGRPESETSCLVTTTDFQIQTEMA ATMETSIFTTEYQVAVAGCVFLLISVLLLSGLTWQRRQRKSRRTIS
Soluble TNFαR	MGLSTVPDLLLPLVLLELLVGIYPSGVIGLVPHLGDREKRDSVCPQG KYIHPQNNSICCTKCHKGTYLYNDCPGPGQDTDCRECESGSFTASEN HLRHCLSCSKCRKEMGQVEISSCTVDRDTVCGCRKNQYRHYWSENL FQCFNCSLCLNGTVHLSCQEKQNTVCTCHAGFFLRENECVSCSNCKK SLECTKLCLPQIENVKGTEDSGTTLVPRGSEPKSCDKTHTCPPCPAPEL LGGPSVFLFPPKPKDTLMISRTPEVTCVVVDVSHEDPEVKFNWYVDG VEVHNAKTKPREEQYNSTYRVVSVLTVLHQDWLNGKEYKCKVSNK ALPAPIEKTISKAKGQPREPQVYTLPPSREEMTKNQVSLTCLVKGFYP SDIAVEWESNGQPENNYKTTPPVLDSDGSFFLYSKLTVDKSRWQQGN VFSCSVMHEALHNHYTQKSLSLSPGK
hPD-L1	MRIFAVFIFMTYWHLLNAFTVTVPKDLYVVEYGSNMTIECKFPVEKQ LDLAALIVYWEMEDKNIIQFVHGEEDLKVQHSSYRQRARLLKDQLSL GNAALQITDVKLQDAGVYRCMISYGGADYKRITVKVNAPYNKINQRI LVVDPVTSEHELTCQAEGYPKAEVIWTSSDHQVLSGKTTTTNSKREEK LFNVTSTLRINTTTNEIFYCTFRRLDPEENHTAELVIPELPLAHPPNERTH LVILGAILLCLGVALTFIFRLRKGRMMDVKKCGIQDTNSKKQSDTHLEET
hCTLA4	MACLGFQRHKAQLNLATRTWPCTLLFFLLFIPVFCKAMHVAQPAVVLA SSRGIASFVCEYASPGKATEVRVTVLRQADSQVTEVCAATYMMGNELT FLDDSICTGTSSGNQVNLTIQGLRAMDTGLYICKVELMYPPPYYLGIGNG TQIYVIDPEPCPDSDFLLWILAAVSSGLFFYSFLLTAVSLSKMLKKRSPLT TGVYVKMPPTEPECEKQFQPYFIPIN
hCD39	MEDTKESNVKTFCSKNILAILGFSSIIAVIALLAVGLTQNKALPENVKY GIVLDAGSSHTSLYIYKWPAEKENDTGVVHQVEECRVKGPGISKFVQ KVNEIGIYLTDCMERAREVIPRSQHQETPVYLGATAGMRLLRMESEEL ADRVLDVVERSLSNYPFDFQGARIITGQEEGAYGWITINYLLGKFSQK TRWFSIVPYETNNQETFGALDLGGASTQVTFVPQNQTIESPDNALQFR LYGKDYNVYTHSFLCYGKDQALWQKLAKDIQVASNEILRDPCFHPG YKKVVNVSDLYKTPCTKRFEMTLPFQQFEIQGIGNYQQCHQSILELFN TSYCPYSQCAFNGIFLPPLQGDFGAFSAFYFVMKFLNLTSEKVSQEKV TEMMKKFCAQPWEEIKTSYAGVKEKYLSEYCFSGTYILSLLLQGYHF TADSWEHIHFIGKIQGSDAGWTLGYMLNLTNMIPAEQPLSTPLSHSTY VFLMVLFSLVLFTVAIIGLLIFHKPSYFWKDMV
hIL-2	MYRMQLLSCIALSLALVTNSAPTSSSTKKTQLQLEHLLLDLQMILNGI NNYKNPKLTRMLTFKFYMPKKATELKHLQCLEEELKPLEEVLNLAQS KNFHLRPRDLISNINVIVLELKGSETTFMCEYADETATIVEFLNRWITF CQSIISTLT

Table S3.

List of antibody and clones.

Antigen	Clone	Supplier
CD25	4E3	Thermo Scientific
TGFβ1	TW4-9E7	BD Biosciences
pSTAT5	SRBCZX	Thermo Scientific
Insulin	EPR17359	Abcam
CD19	HIB19	Biolegend
DNA	3519	Abcam

IFNγ	IFNG/466	Abcam
CD45	HI30	Biolegend
CD4	SK3	Biolegend
CD8	SK1	BD Biosciences
CD127	HIL-7R-M21	BD Biosciences
FoxP3	236A/E7	Thermo Scientific
Helios	22F6	Thermo Scientific
CD3	OKT3	Thermo Scientific
CD28	CD28.2	Thermo Scientific
Myc-Tag	9B11	Cell Signaling
V5-Tag	R963-25	Thermo Scientific
Ki67	SolA15	Thermo Scientific
TNFα	MAb11	Thermo Scientific
Granzyme B	GB11	Thermo Scientific
IL-2	MQ1-17H12	BioLegend

Fig. S1. Analysis of synthetic suppressor cell payload production and differential payload affects on CD4+ vs CD8+ CAR T cells (Related to Fig 1).

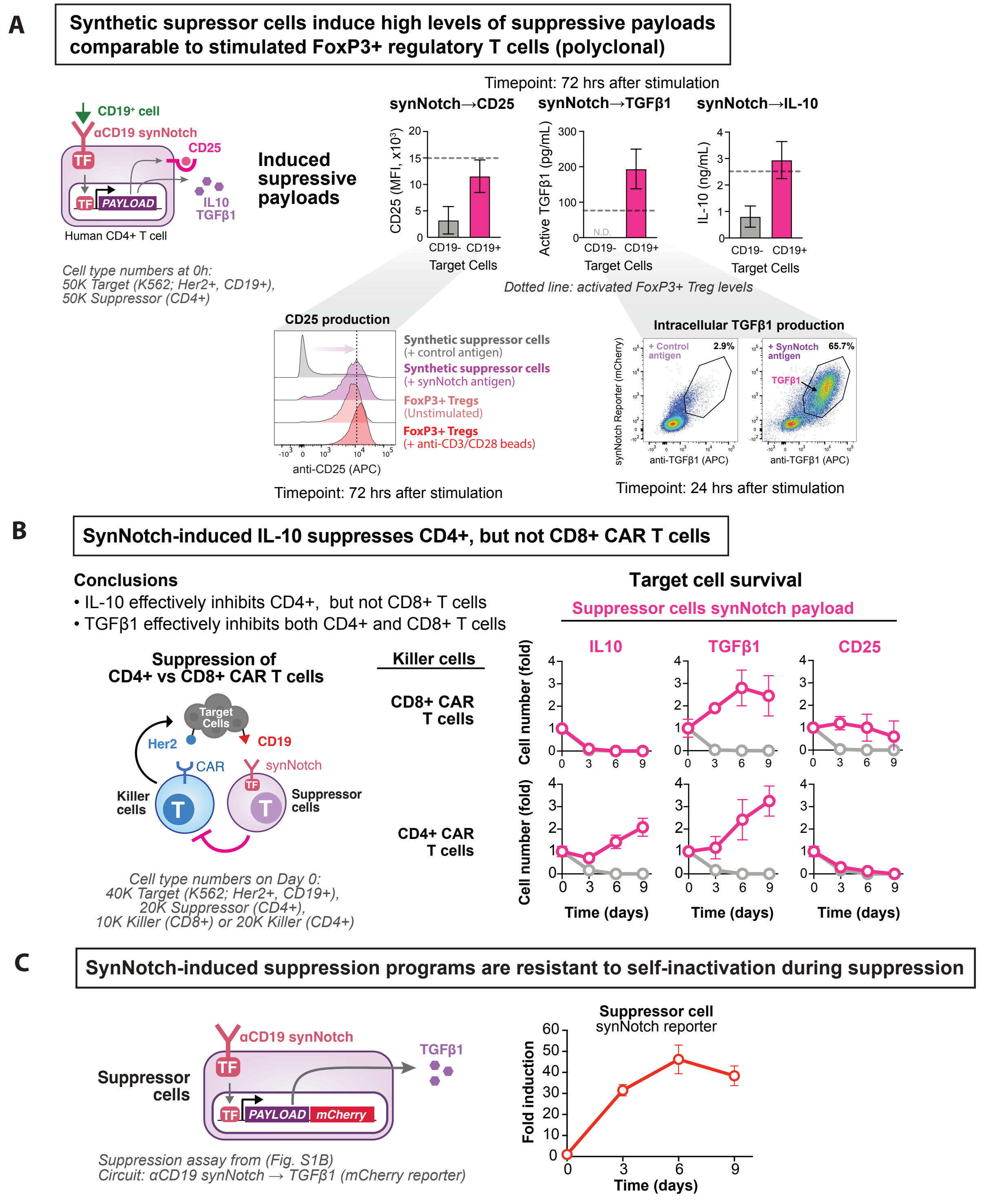

Fig. S1. Analysis of synthetic suppressor cell payload production and differential payload effects on CD4+ vs CD8+ CAR T cells (Related to Fig 1).
(A) SynNotch-induced payloads were produced at high levels comparable to stimulated human polyclonal regulatory T cells. CD25 was measured by antibody staining after co-culture with target cells after 72 hours. IL-10 and TGFβ1 was measured by ELISA of supernatant after co-culture with target cells after 72 hours (n=3, error bars = standard error). Intracellular staining shows TGFβ1 was only produced by suppressor T cells when co-cultured with synNotch antigen positive target cells (K562; CD19+), but not control target cells after 24 hours of co-culture.
(B) SynNotch-induced IL-10 suppressed CD4+ but not CD8+ CAR T cell killing, while TGFβ1 suppresses both CD4+ and CD8+ CAR T cells. Three cell co-culture in vitro (CAR T cells, suppressor cells, and target cells) were used to test suppression as described in (*Fig. 1A*). Target cell survival (cell number - fold) was measured by flow cytometry and normalized to the day 0 timepoint (n=3, error bars = standard error). Grey line indicates the no suppressor T cell control.
(C) SynNotch induction circuits are not self-inactivated by production of immunosuppressive signals. For suppression assay in (*B*), suppressor T cells producing TGFβ1 maintained synNotch reporter (mCherry) expression during suppression (n=3, error bars = standard error).

Fig. S2. Analysis of synthetic suppressor cell inducing combinatorial payloads to suppress CAR CD4+ and CAR CD8+ T cells (Related to Fig 1).

Synthetic suppressor cells inducing a combination of CD25 with an inhibitory payload (IL-10, TGFβ1, PD-L1) leads to most potent suppression of CD4+ and CD8+ CAR T cells *in vitro*.

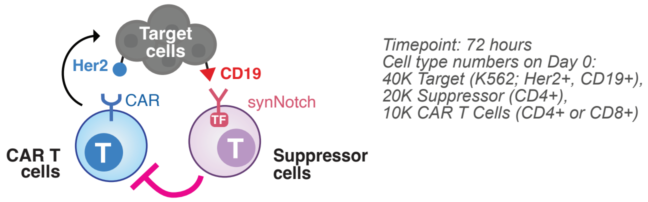

Circuit: anti-CD19 synNotch → induced payload 1 + induced payload 2

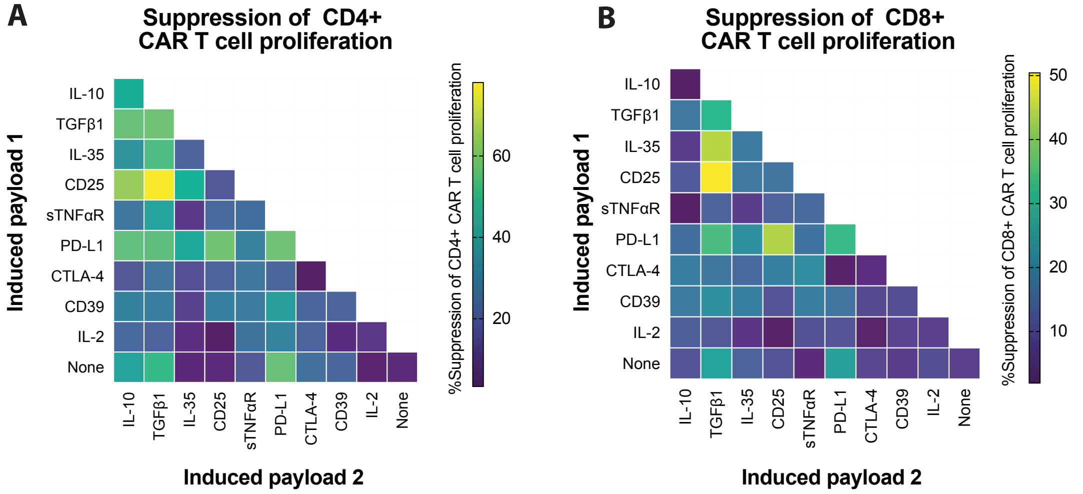

Fig. S2. Analysis of synthetic suppressor cell inducing combinatorial payloads to suppress CAR CD4+ and CAR CD8+ T cells (Related to Fig 1).
(A) Suppression of primary CD4+ anti-Her2 CAR T cells in co-culture with primary CD4+ synthetic suppressor T cells inducing two payloads. The percent suppression of CAR T cell proliferation (normalized to no suppressor control) is shown. Cell counts measured by flow cytometry at 72 hours (mean, n=3).
(B) Suppression of primary CD8+ anti-Her2 CAR T cells in co-culture with primary CD4+ synthetic suppressor T cells inducing two payloads. The percent suppression of CAR T cell proliferation shown as in (*A*) and counts were measured after 72 hours (mean, n=3).

Fig. S3. Analysis of synthetic suppressor cell inducing combinatorial payloads to suppress stimulated polyclonal CD4+ and CD8+ T cells (Related to Fig 1).

Synthetic suppressor cells inducing a combination of CD25 with an inhibitory payload leads to most potent suppression of stimulated polyclonal CD4+ and CD8+ T cells *in vitro*.

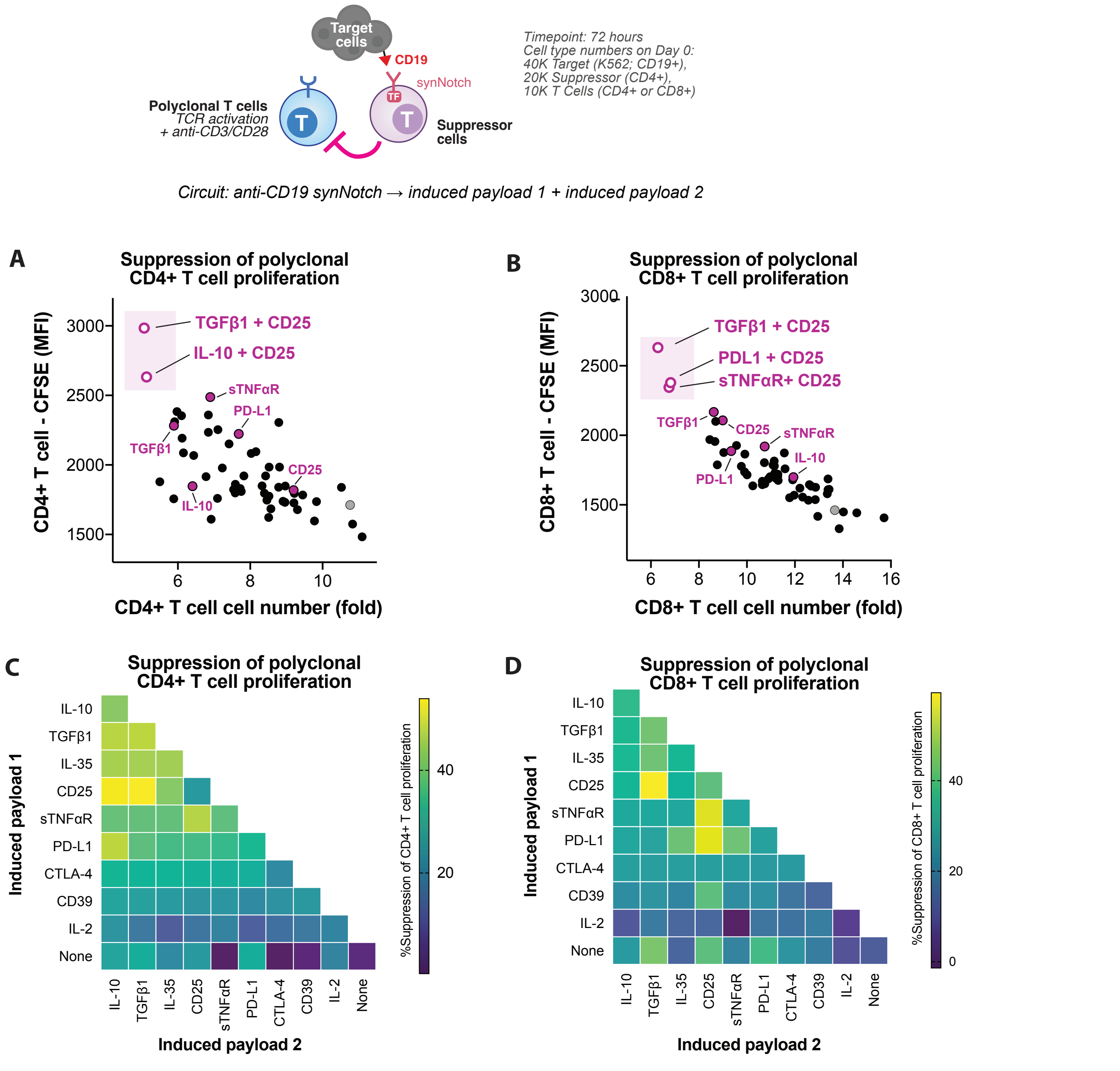

Fig. S3. Analysis of synthetic suppressor cell inducing combinatorial payloads to suppress stimulated polyclonal CD4+ and CD8+ T cells (Related to Fig 1).

(A) Combinations of synNotch-induced payloads drive stronger suppression of polyclonal CD4+ T cells *in vitro*. Suppression of human CD4+ T cells (pre-activated by anti-CD3/CD28 antibody for 24 hours) in co-culture with synthetic suppressor T cells inducing two payloads and target cells. The fold proliferation was measured by dilution of CFSE CellTrace label (MFI) of CD4+ T cells and cell number fold change over 72 hours. Each point indicates a pairwise combination of payloads from the library in (*Fig 1A*) induced by anti-CD19 synNotch suppressor cells (mean, n=3). Fold change normalized to the 0 hour timepoint. Grey point indicates the no payload suppressor T cell control.

(B) Combinations of payloads drive stronger suppression of polyclonal CD8+ T cells *in vitro*. Same as in (*A*) with CD8+ T cells.

(C) Suppression of polyclonal CD4+T cells by combinatorial payloads, same as in (*A*).The percent suppression of T cell proliferation (normalized to no suppressor control cell number) is shown. Cell counts measured by flow cytometry at 72 hours (mean, n=3).

(D) Suppression of polyclonal CD8+T cells by combinatorial payloads, same as in (*B*). T cell proliferation shown as in (*C*).

Fig. S4. Additional analysis of combination payload suppressor cells (synNotch → [IL-10 + CD25]; or synNotch → [TGFβ1+ CD25]) (Related to Fig 2).

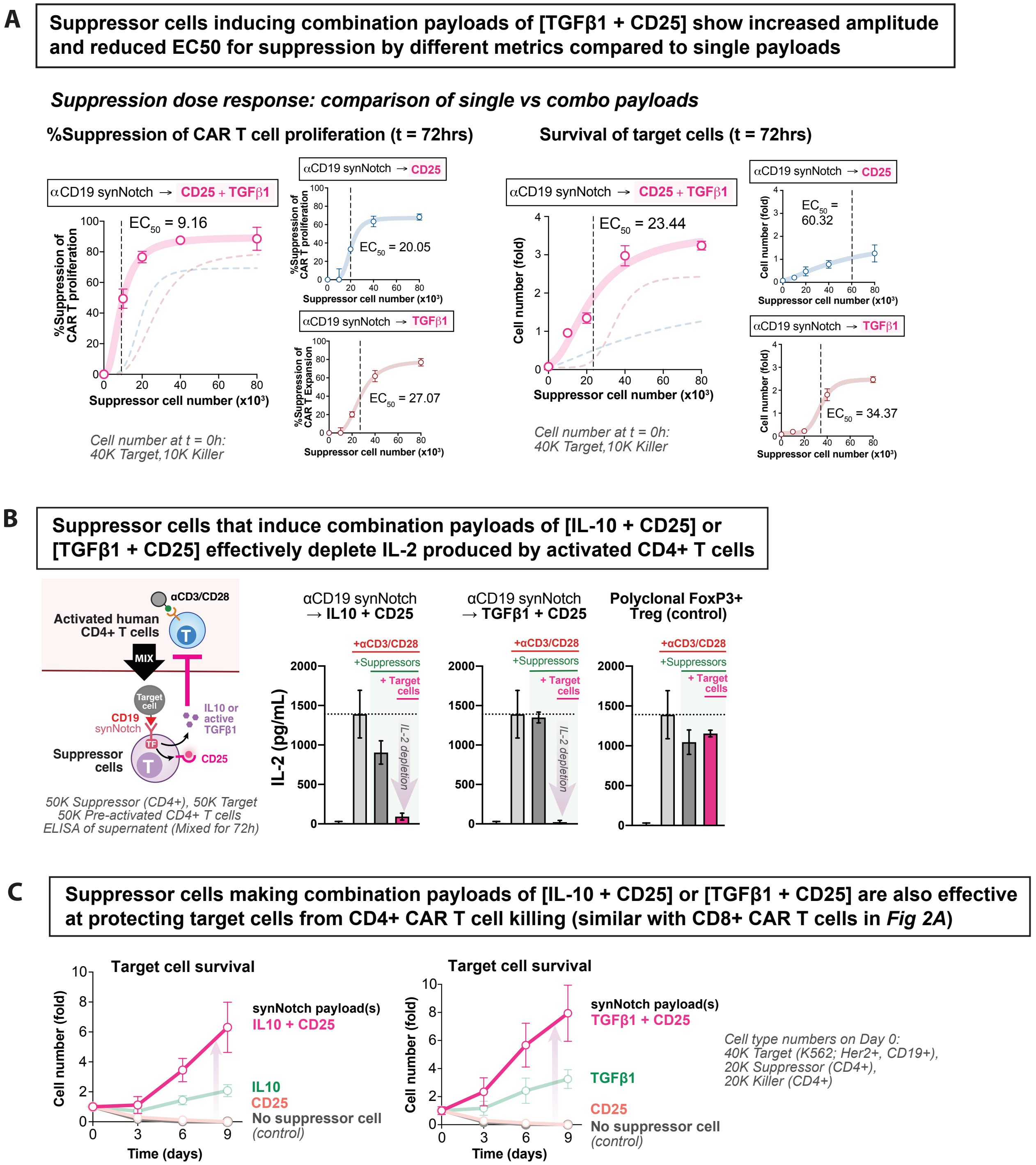

Fig. S4. Additional analysis of combination payload suppressor cells (synNotch → [IL-10 + CD25]; or synNotch → [TGFβ1+ CD25]) (Related to Fig 2).
(A) Suppression of CD8+ CAR T cell killing and proliferation as a dose response of synthetic suppressor T cell number show that combinatorial circuits (anti-CD19 synNotch → TGFβ1+CD25) increase the amplitude and reduce the EC50 for suppression compared to individual payloads. The percent suppression of CAR T cell proliferation (normalized to no suppressor control) is shown, t = 72h (n=6, error bars = standard error).
(B) SynNotch-induced payloads block accumulation of IL-2 produced by activated CD4+ T cells. Human CD4+ T cells were pre-activated for 24 hours using anti-CD3/CD28 beads prior to co-culturing with anti-CD19 synthetic suppressor T cells activated by CD19+ target cells. IL-2 levels in the supernatant were measured by ELISA after 72 hours of co-culture. Polyclonal FoxP3+ Tregs were pre-activated with anti-CD3/CD28 beads for 24 hours prior to co-culture, showing limited antigen-specific suppression of IL-2 secretion (n=3, error bars = standard error).
(C) Synthetic suppressor T cells with synNotch → IL-10+CD25 or synNotch → TGFβ1+CD25 circuit block CAR CD4+ T cell killing of target cells more effectively than each payload alone. Target cell survival measured by flow cytometry and normalized to the day 0 timepoint (n=3, error bars = standard error).

Fig. S5. Additional analysis of synthetic suppressor cell proliferation, IL-2 consumption, and signaling (Related to Fig 2).

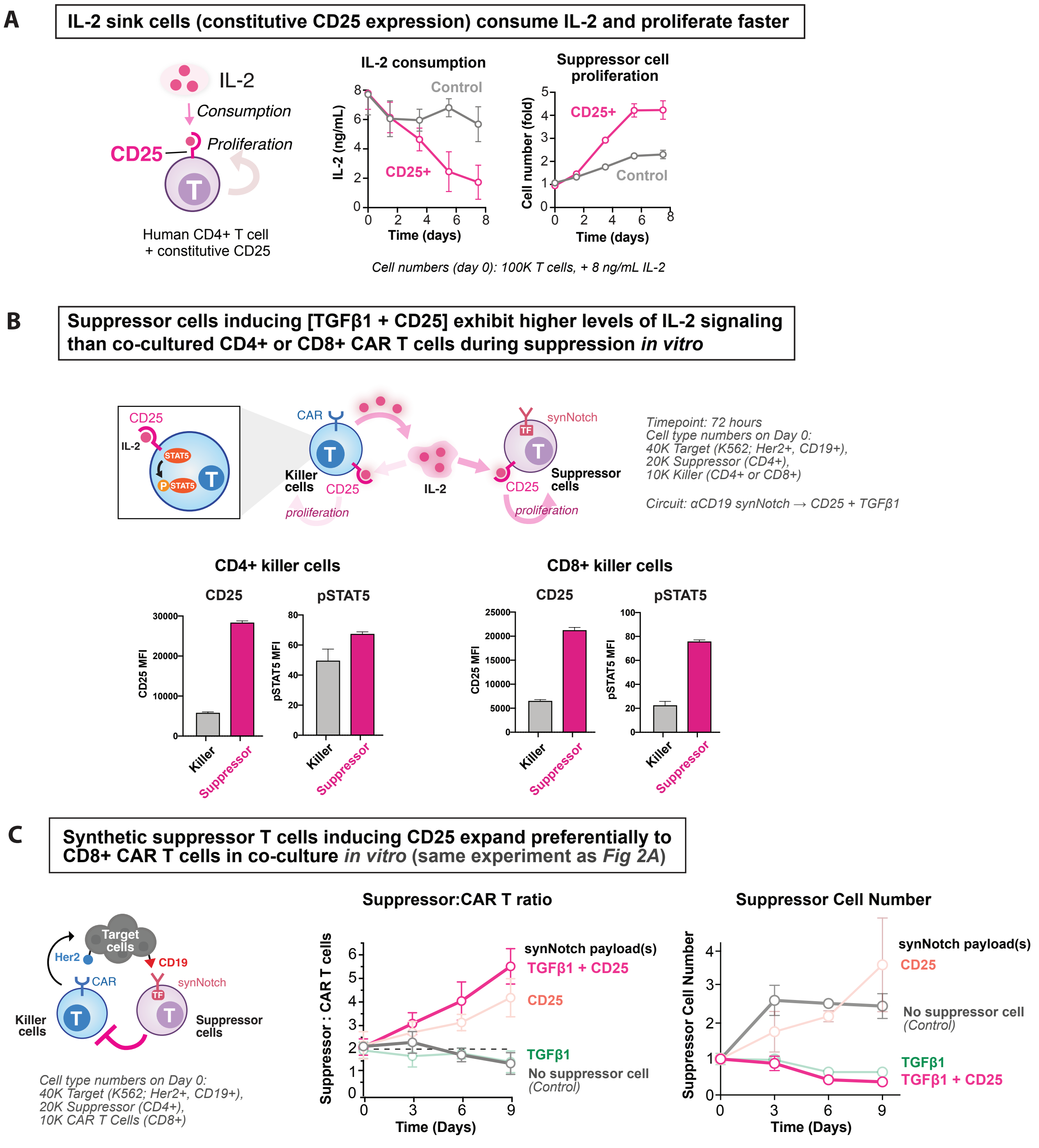

Fig. S5. Additional analysis of synthetic suppressor cell proliferation, IL-2 consumption, and signaling (Related to Fig 2).
(A) Engineered CD25+ CD4+ T cells consume IL-2 from the media and proliferate faster. Cell counts were measured by flow cytometry (normalized to day 0 counts) and IL-2 levels measured by ELISA of supernatant (n=3, error bars = standard error).
(B) Synthetic suppressor T cells with anti-CD19 synNotch → CD25+TGFβ1 circuit show stronger IL-2 signaling (pSTAT5, CD25) than CD4+ or CD8+ CAR cells in the same co-culture. Suppression assay was performed as described in (*Fig. 1A*). CD25 and pSTAT5 was measured by staining T cells in the same co-culture t=72 hours (n=6, error bars = standard error).
(C) Suppressor T cells that express CD25 expand preferentially to CAR T cells during suppression *in vitro*. Synthetic suppressor cell counts relative to CAR T cell counts over time and suppressor T cell cell numbers over time (normalized to day 0 counts) are shown for experiment in *Fig 2A* (n=3, error bars = standard error).

Fig. S6. Additional analysis of CAR T cell activation and pro-inflammatory cytokine secretion (Related to Fig 2).

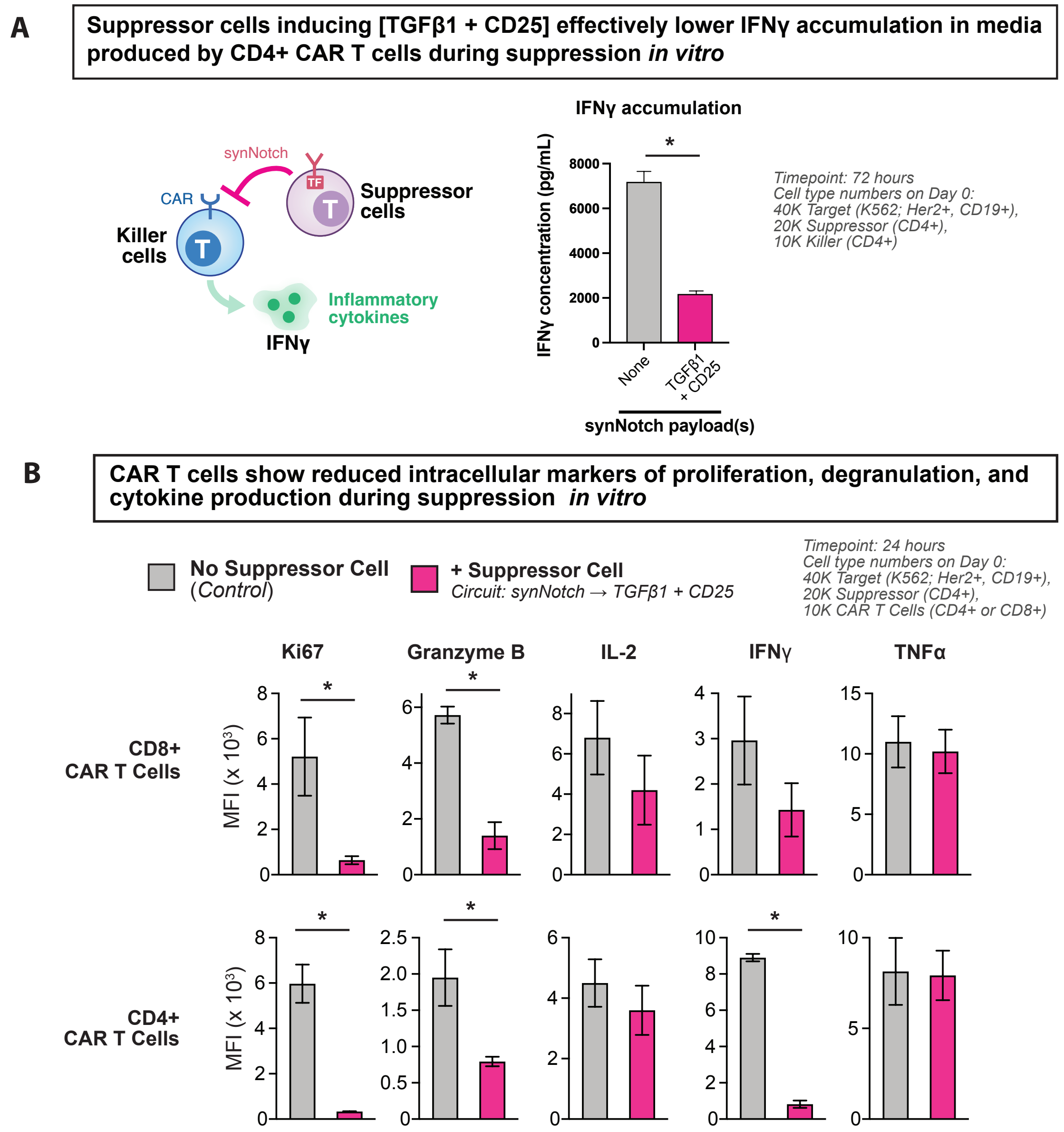

Fig. S6. Additional analysis of CAR T cell activation and pro-inflammatory cytokine secretion (Related to Fig 2).
(A) Synthetic suppressor T cells with anti-CD19 synNotch→ CD25+TGFβ1 lower IFNγ accumulation in media *in vitro*. As in (*B*), T cells were co-cultured with target cells. Media was collected after 72 hours and IFNγ levels were measured by ELISA (n=3, error bars = standard error, two-tailed t test between groups *P < 0.05).
(**B**) CAR T cells show lower levels of proliferation, degranulation, and cytokine production during suppression *in vitro*. CAR T cells were stained for intracellular markers after 24 hours in suppresesion assay co-culture (n=3, error bars = standard error, two-tailed t test between groups *P < 0.05).

Figure S7. Individual mouse tumor growth in two-tumor model (Related to Fig 3).

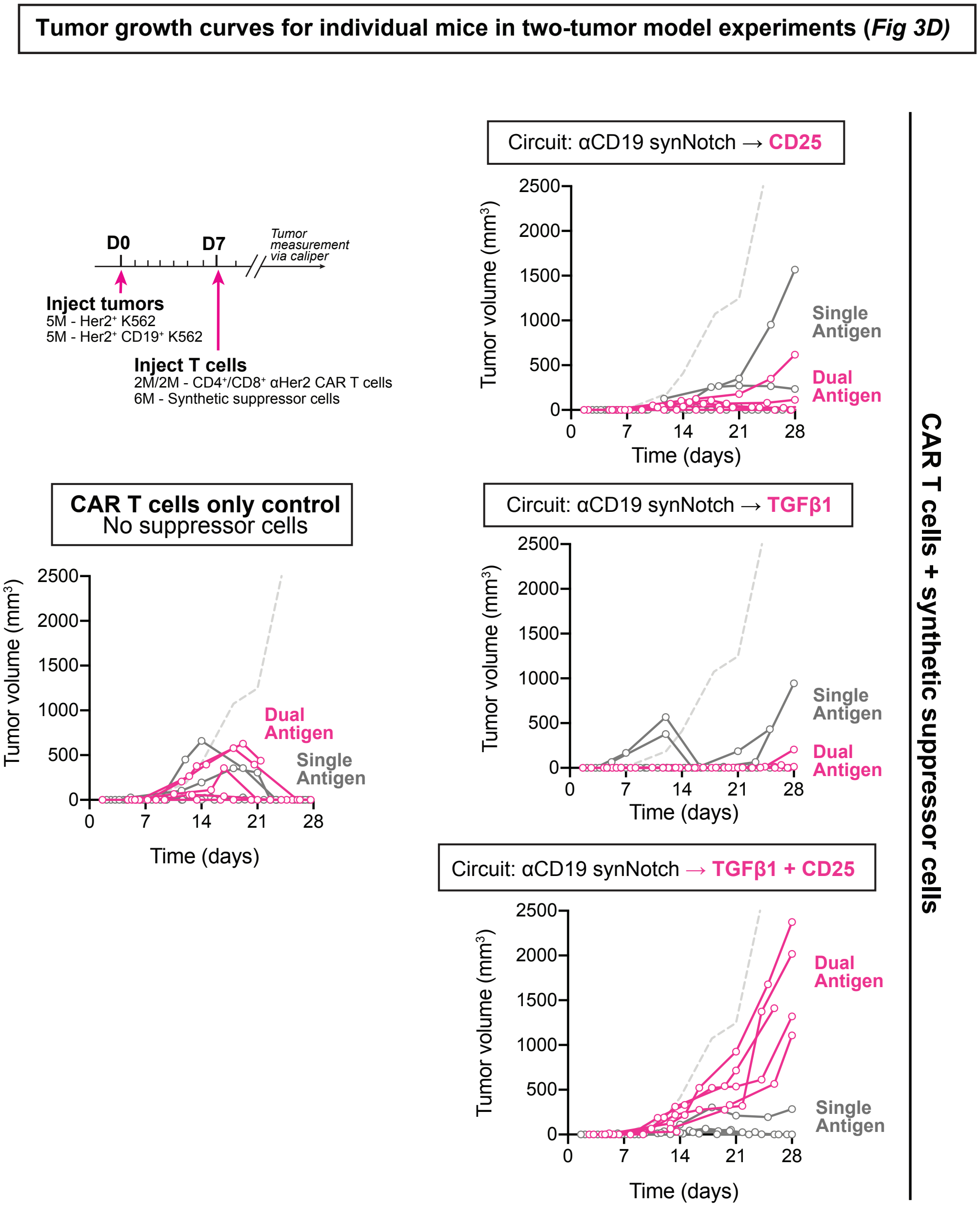

Figure S7. Individual mouse tumor growth in two-tumor model (Related to Fig 3). Local suppression of CAR T cell killing by synthetic suppressor T cells in the two-tumor model (dual antigen tumor protection without compromising killing of the single antigen tumor) with different payloads (showing invidual mice from *Fig 3B*). Individual mouse tumors measured by caliper. Dashed line indicates tumor growth with no T cell injection.

Fig. S8. Comparison of synthetic suppressor T cells to FoxP3+ polyclonal and CAR-engineered Tregs *in vitro* and *in vivo* (Related to Fig 3).

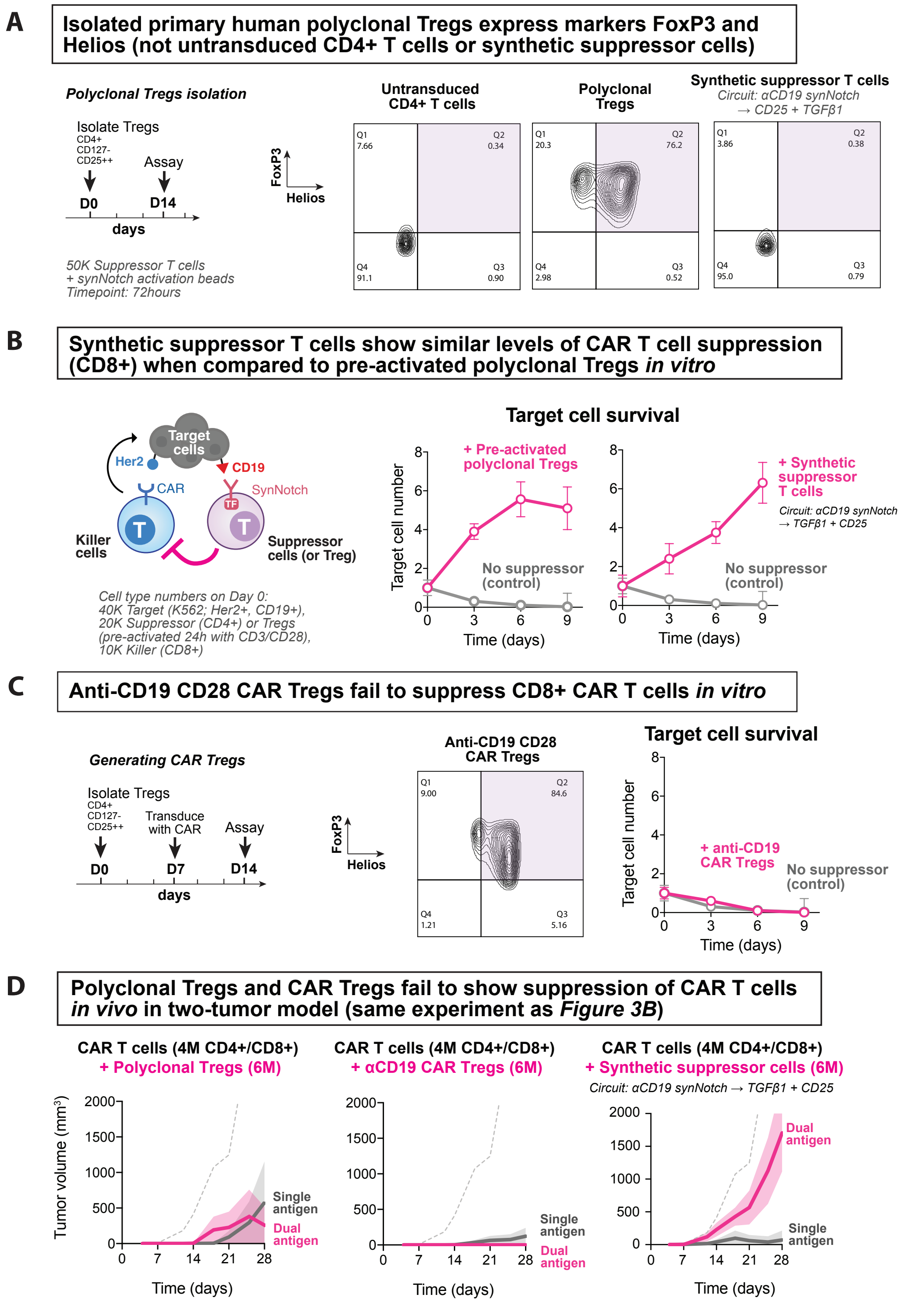

Fig. S8. Comparison of synthetic suppressor T cells to FoxP3+ polyclonal and CAR-engineered Tregs *in vitro* and *in vivo* (Related to Fig 3).
(A) Isolation and staining of human polyclonal FoxP3+ Tregs. FoxP3+ Tregs were isolated by sorting CD4+ CD127- CD25++ and stained for markers FoxP3 and Helios at day 14 after isolation. Untransduced CD4+ T cells or synthetic suppressor cells (activated for 72 hours with anti-Myc beads) do not show any FoxP3+ or Helios+ population.
(B) Synthetic suppressor cells and polyclonal Tregs (pre-activated for 24 hours with anti-CD3/CD28 antibody) showed similar suppression of CD8+ CAR T cell killing *in vitro*. Suppression assay co-culture was performed as previously described (*Fig 1A*) and cell counts (normalized to the day 0 timepoint) were determined by flow cytometry (n=3, error bars = standard error)
(C) CAR Tregs failed to show suppression of CD8+ CAR T cells *in vitro*. Suppression assay was performed as in (*B*) using anti-CD19 CAR Tregs with CD28 co-stimulatory domain (n=3, error bars = standard error).
(D) FoxP3+ polyclonal and CAR Tregs didnot show local suppression *in vivo* in two-tumor model. Polyclonal Tregs and anti-CD19 CAR Tregs were co-injected with cytotoxic CD4+ and CD8+ CAR Tregs as in (*Fig 3B*, same experiment), but fail to show local suppression of cytotoxic CAR T cell killing. (n=3, solid line = mean, shading = standard error). Dashed grey line indicates tumor growth with no T cell injection.

Figure S9. Replicates and individual mice of NOT gate circuit with different human T cell donors (Related to Fig 3).

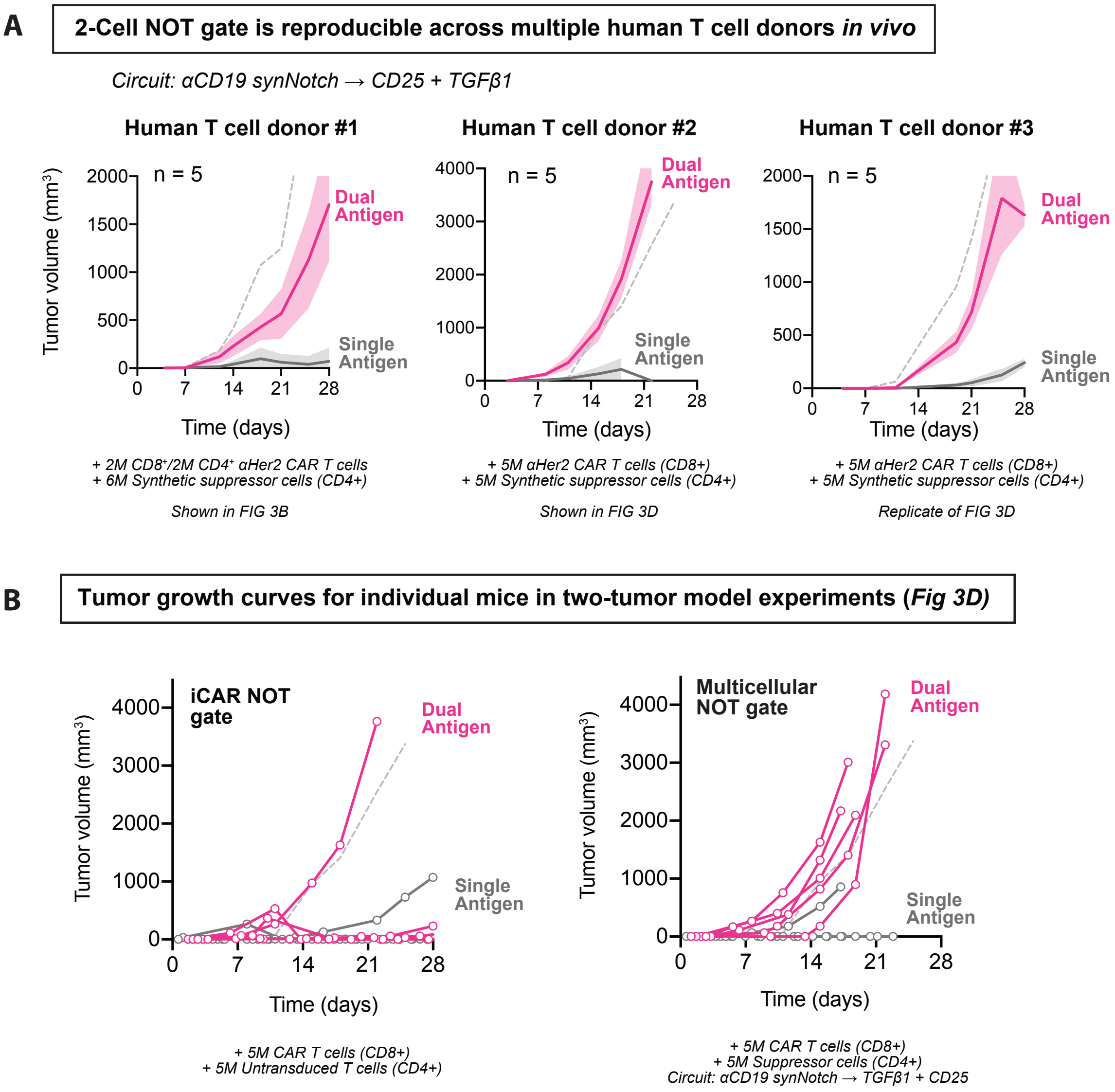

Figure S9. Replicates and individual mice of NOT gate circuit with different human T cell donors (Related to Fig 3).
(**A**) Two-cell NOT gate tumor killing circuit was tested as in (*Fig. 3B, D*) using different human T cell donor (n=3 to 5, solid line = mean, shading = standard error). Circuits using all 3 human T cell donors show local suppression of CAR T cell killing in the dual antigen tumor without compromising killing of the single antigen tumor. Dashed line indicates tumor growth with no T cell injection.
(**B**) Individual mice shown for *Fig 3D*. Individual mouse tumors measured by caliper. Dashed line indicates tumor growth with no T cell injection.

Fig. S10. Synthetic suppressor T cells protect bystander cells, overcoming heterogeneous synNotch priming antigen (Related to Fig 3).

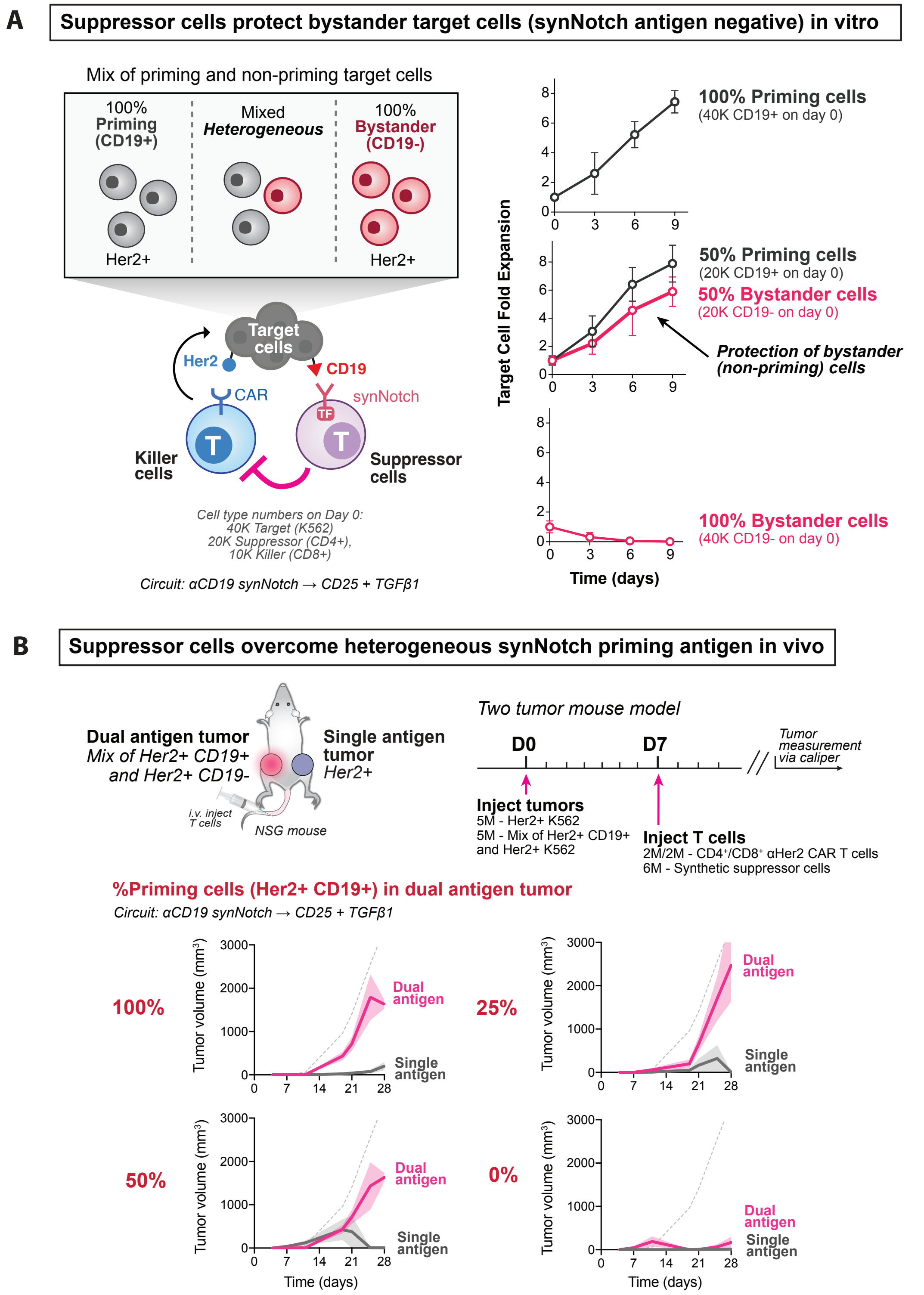

Fig. S10. Synthetic suppressor T cells protect bystander cells, overcoming heterogeneous synNotch priming antigen (Related to Fig 3).
(A) Synthetic suppressor T cells block killing of bystander target cells (synNotch antigen negative) by CAR T cells. Target cells (Her2+) with or without the synNotch antigen, CD19, were mixed at a 1:1 ratio at day 0. Cell counts were measured by flow cytometry and normalized to the day 0 timepoint. Bystander target cells (GFP+) and target cells with CD19 antigen (BFP+) were tracked by fluorescent labels (n = 3, error bars = standard error).
(B) Synthetic suppressor T cells overcome heterogeneous synNotch priming antigen in vivo. Tumors containing only single antigen target cells (Her2+) or a combination of single and dual antigen (Her2+ CD19+) target cells were injected subcutaneously in NSG mice. Synthetic suppressor T cells block CAR T cell killing of tumors with as low as 25% synNotch priming cells in the tumor at the time of implantation. The single antigen tumor was cleared in all cases. Dashed line indicates tumor growth with no T cell injection (n = 5, solid line = mean, shading = standard error).

Fig. S11. Enriched beta cell clusters engineered to express CD19 are functional and activate synthetic suppressor cells in vitro (Related to Fig 4).

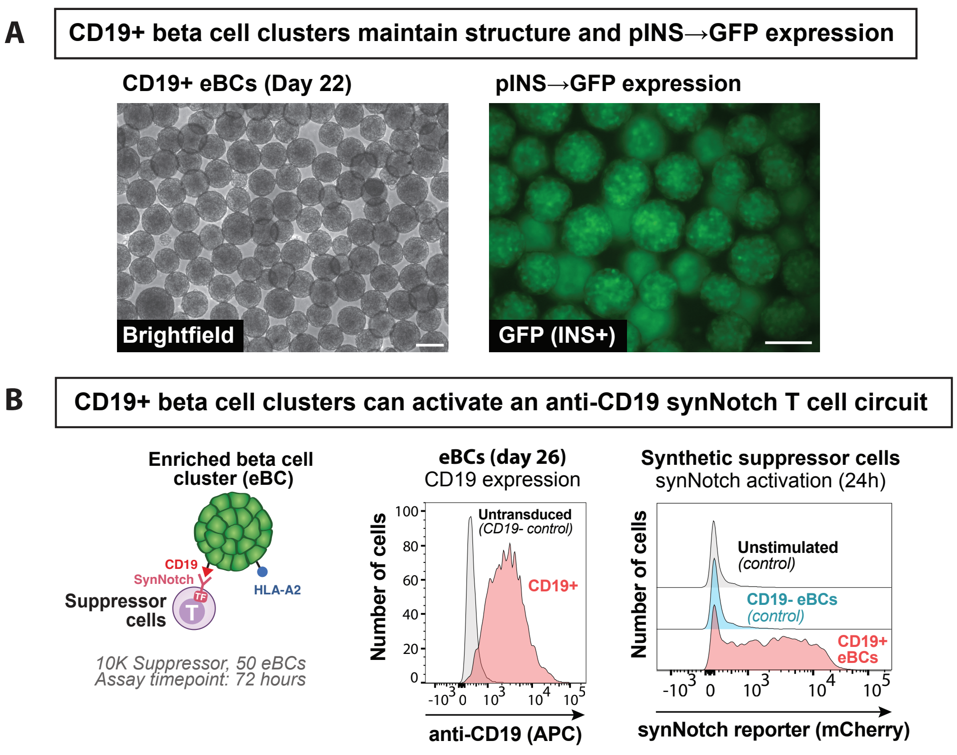

Fig. S11. Enriched beta cell clusters engineered to express CD19 are functional and activate synthetic suppressor cells in vitro (Related to Fig 4).
(A) Human pluripotent stem cell (hPSC)-derived enriched beta cell (eBCs) organoids engineered to express human CD19 model antigen maintain characteristic morphology and insulin promoter induced GFP expression in vitro at day 22 of differentiation. Bars indicates 100 µm.
(B) Anti-CD19 synNotch synthetic suppressor T cells can activate the expression of a synNotch reporter (mCherry) when co-cultured with human CD19+ eBC organoids, but not untransduced eBC organoid controls (CD19 negative). Fluorescence measured by staining for human CD19 and flow cytometry after 24 hours of co-culture.

Fig. S12. Analysis of hPSC-derived beta cell transplants (Related to Fig 5).

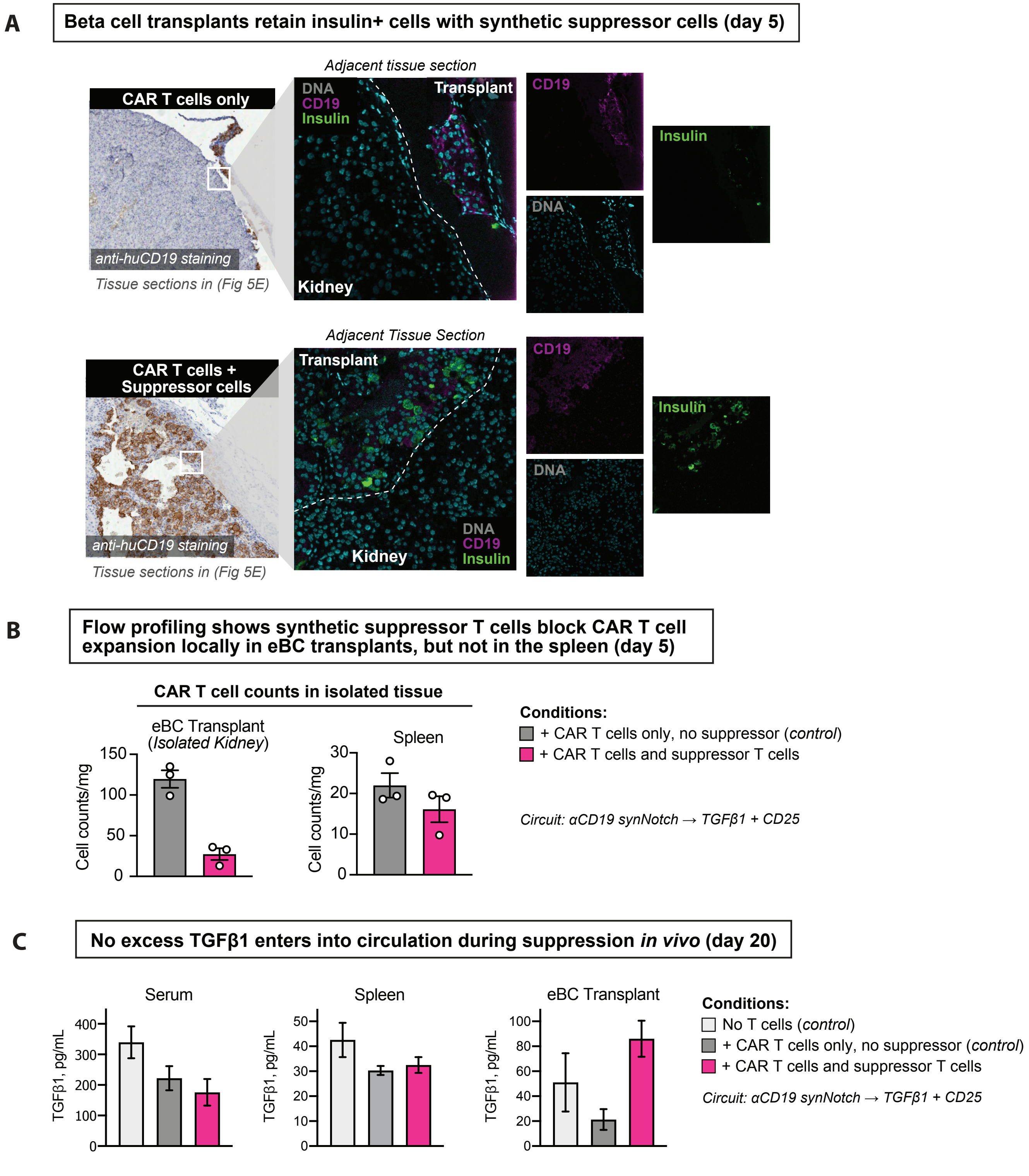

Fig. S12. Analysis of hPSC-derived beta cell transplants (Related to Fig 5).
(A) Multiplex Ion Beam Imaging (MIBIscope) analysis of transplanted eBC organoids (isolated 5 days after T cell injection) show higher insulin staining with synthetic suppressor T cells (anti-CD19 synNotch→ CD25+TGFβ1) compared to CAR T cells alone. Adjacent tissue sections from the anti-huCD19 staining in (*Fig 5E*) were used.
(B) Flow profiling of isolated kidneys and spleens (5 days after T cell injection) show significant reduction in CD4+/CD8+ CAR T cell counts in the kidney (site of eBC organoid transplantation), but no significant difference in the number of CAR T cells in the spleen. Error bars indicate standard error.
(**C**) TGFβ1 produced by suppressor T cells does not cause excess acculumation of TGFβ1 in circulation. CD19+ eBC organoid transplants were performed as in (*Fig 5A*). Blood, spleen, and transplants were collected at day 20 after T cell injection and levels of TGFβ1 were masured by ELISA (mean, n=5, error bars = standard error).
